# Excited State Assignment and State‐Resolved Photoelectron Circular Dichroism in Chalcogen‐Substituted Fenchones

**DOI:** 10.1002/cphc.202500319

**Published:** 2025-11-03

**Authors:** Sudheendran Vasudevan, Steffen M. Giesen, Simon T. Ranecky, Lutz Marder, Igor Vidanović, Manjinder Kour, Catmarna Küstner‐Wetekam, Nicolas Ladda, Sagnik Das, Tonio Rosen, Vidana Popkova, Han‐gyeol Lee, Denis Kargin, Tim Schäfer, Andreas Hans, Thomas Baumert, Robert Berger, Hendrike Braun, Arno Ehresmann, Guido W. Fuchs, Thomas F. Giesen, Jochen Mikosch, Rudolf Pietschnig, Arne Senftleben

**Affiliations:** ^1^ Institut für Physik and CINSaT Universität Kassel Heinrich‐Plett‐Str. 40 34132 Kassel Germany; ^2^ Fachbereich Chemie Philipps Universität Marburg Hans‐Meerwein Str. 4 35032 Marburg Germany; ^3^ Institut für Chemie and CINSaT Universität Kassel Heinrich‐Plett‐Str. 40 34132 Kassel Germany; ^4^ Institut für Physikalische Chemie Georg‐August‐Universität Göttingen Tammannstr. 6 37077 Göttingen Germany; ^5^ Institut für Physik Universität Kassel Heinrich‐Plett‐Str. 40 34132 Kassel Germany

**Keywords:** chirality, circular dichroism, femtochemistry, light‐matter interactions, photoelectron spectroscopy

## Abstract

Excited electronic states of fenchone, thiofenchone, and selenofenchone are characterized and assigned with different gas‐phase spectroscopic methods and ab initio quantum chemical calculations. With an increasing atomic number of the chalcogen, increasing bathochromic (red) shifts are observed, which vary in strength for Rydberg states, valence‐excited states, and ionization energies. The spectroscopic insight is used to state‐resolve the contributions in multiphoton photoelectron circular dichroism with femtosecond laser pulses. This is shown to be a sensitive observable of molecular chirality in all studied chalcogenofenchones. This work contributes new spectroscopic information, particularly on thiofenchone and selenofenchone. It may open a perspective for future coherent control experiments exploiting resonances in the visible and near‐ultraviolet spectral regions.

## Graphical abstract

Fenchone, thiofenchone, and selenofenchone (chalcogenofenchones) are characterized using gas‐phase spectroscopic experiments. The experimentally determined energies of excited states are supported by quantum chemical calculations. Furthermore, near‐ultraviolet femtosecond laser pulses are used to measure the state‐resolved photoelectron circular dichroism of both enantiomers.

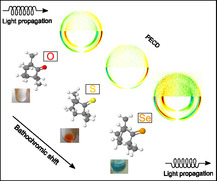

## 1 Introduction

Sensing the chirality of molecules in the gas phase has become a popular scientific frontier. Taking advantage of the interaction‐free environment, coherent microwave spectroscopy,^[^ ^1^, ^2^, ^3^ ^]^ Coulomb explosion imaging,^[^ ^4^, ^5^, ^6^, ^7^ ^]^ and circular dichroism (CD) using high harmonic spectroscopy^[^ ^8^ ^]^ or ion‐yield^[^ ^9^, ^10^, ^11^, ^12^, ^13^, ^14^, ^15^ ^]^ have been demonstrated in the last decade. Moreover, photoelectron circular dichroism (PECD) has been established as a versatile probe of chirality.^[^ ^16^ ^]^ PECD is a forward/backward asymmetry in the angular distribution of electrons derived from randomly oriented chiral molecules by ionization with circularly polarized light. It results from the scattering of the departing photoelectron by the potential created by the atomic cores of the molecule. The asymmetry in the photoelectron angular distribution changes sign either by changing the enantiomer or the helicity of the circularly polarized light. The strength of the asymmetry can be measured by the linear PECD (LPECD) quantity.^[^ ^17^, ^18^ ^]^ Since PECD is attributed purely to electric dipole interaction,^[^ ^19^ ^]^ it exhibits a stronger effect^[^ ^20^ ^]^ than other chiroptic effects based on electric quadrupole or magnetic dipole interaction such as CD.^[^ ^13^, ^14^, ^21^ ^]^

After the theoretical prediction by Ritchie in 1976,^[^ ^19^ ^]^ it took more than 25 years before the first experimental observation of PECD was reported using synchrotron radiation.^[^ ^22^ ^]^ For ionization with extreme ultraviolet (XUV) and X‐ray photons, the scattering electron that probes the ionic potential can originate either from core shells^[^ ^23^, ^24^, ^25^, ^26^ ^]^ or valence shells^[^ ^26^, ^27^, ^28^, ^29^, ^30^, ^31^, ^32^ ^]^ of the molecule, depending on the photon energy.

**Figure 1:**
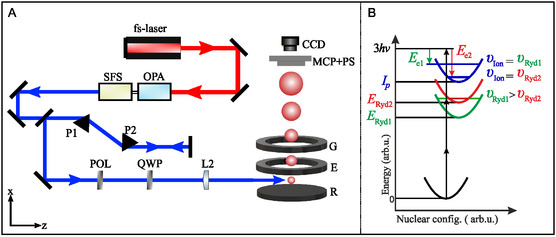
A) Experimental setup for femtosecond REMPI‐PECD. It consists of a titanium‐sapphire femtosecond amplifier, an OPA with sum‐frequency conversion stage (OPA/SFS), a prism compressor (P1, P2) for pulse compression, a nanowire grid polarizer (POL) and a quarter‐wave plate (QWP) to create circularly polarized light, a UV fused silica plano‐convex lens (L2), the VMI electrodes repeller (R), extractor (E), and ground (G), a MCP detector, phosphor screen (PS), and camera (CCD). In the VMI apparatus, the laser pulses propagate along the *z*‐axis, while the particles are accelerated along the *x*‐axis. B) Schematic of the 2 + 1 REMPI processes involving two electronic Rydberg states at energies *E*_Ryd1_ and *E*_Ryd2_. First, two‐photon excitation populates these states at different vibrational states *v*_Ryd1_ > *v*_Ryd2_. Due to the Rydberg nature, the following ionization by another photon hardly changes the vibrational excitation, which gives rise to higher photoelectron energy *E*_*e*2_ from the higher‐lying *E*_Ryd2_ Rydberg state.

Pioneering experiments in the optical domain, reported a decade later, showed that the PECD effect can be equally strong using resonance‐enhanced multiphoton ionization (REMPI) with femtosecond^[^ ^17^, ^33^ ^]^ and nanosecond^[^ ^34^ ^]^ lasers. The principle of REMPI for the example of a 2 + 1 scheme is sketched in **Figure** **1**B, where two photons are necessary to reach a bound excited state as an intermediate resonance, and one additional photon ionizes the molecules. The existence of the intermediate resonance increases the multiphoton ionization rate drastically, making REMPI well‐known for its high selectivity and sensitivity.^[^ ^35^ ^]^ REMPI‐PECD experiments hence raise the obvious question: how do excited states of the chiral molecule contribute in detail to the observed PECD effect? This question continues to be of high interest, despite and because of the increasing number of studies utilizing REMPI‐PECD.^[^ ^17^, ^18^, ^33^, ^36^, ^37^, ^38^, ^39^, ^40^, ^41^, ^42^, ^43^, ^44^, ^45^, ^46^, ^47^, ^48^ ^]^ PECD has also been studied in the strong‐field regime, where resonances do not play a role.^[^ ^38^, ^39^, ^49^ ^]^

Fenchone has become a benchmark molecule in experimental and theoretical studies, turning it into the “hydrogen atom of gas‐phase chirality.”^[^ ^34^, ^36^, ^43^, ^50^, ^51^, ^52^, ^53^, ^54^, ^55^, ^56^ ^]^ It serves as a reference for enantiomeric excess determination with different methods.^[^ ^47^, ^57^, ^58^, ^59^ ^]^ In addition, many experimental and theoretical studies to understand its spectroscopic properties^[^ ^60^, ^61^, ^62^, ^63^, ^64^ ^]^ and internal dynamics^[^ ^52^, ^53^, ^65^ ^]^ have been reported. Single‐photon excitation of fenchone requires wavelengths in the ultraviolet. Derivates in which the oxygen atom is replaced with heavier chalcogens exhibit bathochromic (red) shifts in the absorption spectrum, which might make them more accessible for future coherent control experiments with visible wavelengths.

Here, we investigate the excited states and the linear REMPI‐PECD for fenchone and its heavier derivates, thiofenchone (oxygen substituted by sulfur) and selenofenchone (oxygen substituted by selenium). In the terminology of fenchone as the “hydrogen atom of gas‐phase chirality,” the other chalcogenofenchones are its alkali atoms. While both enantiomers of fenchone are readily commercially available, thiofenchones and selenofenchones have to be synthesized. Consequently, much less spectroscopic information on Rydberg states, valence‐excited states, and ionization energies is available as compared to fenchone. To the best of our knowledge, REMPI photoelectron spectroscopy has not been reported for thiofenchone nor any gas‐phase spectroscopy on selenofenchone. For thiofenchone, the ionization energy has been reported,^[^ ^66^ ^]^ and the *n* = 4 Rydberg state and a *π* → π* state have been identified with gas‐phase UV absorption spectroscopy.^[^ ^67^ ^]^ For selenofenchone, CD and UV–VIS absorption spectra in various solvents have been published, identifying a *n* → π* excitation in the visible spectral region.^[^ ^68^, ^69^ ^]^ These solution‐phase studies have compared the UV–VIS absorption bands of selenofenchone with those of thiofenchone and fenchone, observing a bathochromic shift with increasing atomic number of the chalcogen.

This manuscript is organized as follows. First, we describe our methodology. Experimentally, we use single‐photon vacuum ultraviolet (VUV) absorption spectroscopy, nanosecond laser REMPI spectroscopy, and wavelength scanning multiphoton photoelectron spectroscopy using picosecond‐duration laser pulses. In addition, we use angle‐resolved photoelectron spectroscopy to measure REMPI‐PECD using femtosecond laser pulses. As theoretical approaches, we use quantum chemical calculations on the density functional theory (DFT) and coupled cluster (CC) level. Synthesis and sample characterization of thiofenchone and selenofenchone are detailed in an accompanying article.^[^ ^70^ ^]^ For fenchone, thiofenchone, and selenofenchone, we use our combined results from experiment and theory to obtain the adiabatic *I*_*P*_ and assign excited electronic states. Assisted by calculated values for tellurofenchone and polonofenchone, we will discuss the scaling of excited state energies in chalcogenofenchones. In a subsequent section, we discuss femtosecond REMPI‐PECD experiments on fenchone, thiofenchone, and selenofenchone at a fixed wavelength of 376 nm and use the obtained spectroscopic insight to resolve the contributions of individual states.

## 2 Experimental Section

### 2.1 Sample Preparation

The chalcogenofenchones have two stereocenters, but due to the bicyclic structure, only two enantiomers are geometrically possible: (1*R*, 4*S*) and (1*S*, 4*R*). We will refer to the (1*R*, 4*S*)‐enantiomers as (*R*)‐chalcogenofenchone and to the (1*S*, 4*R*) as (*S*) while also giving the sign of the optical rotation. Commercially available (*S*)‐(+)‐fenchone (*Acros*, purity ≥ 97%, enantiomeric excess (e.e.) ≈ 99%) and (*R*)‐(−)‐fenchone (*Merck*, purity ≥ 98%, e.e. ≈ 84%) were used without further processing. The synthesis and characterization of thiofenchone and selenofenchone will be described in detail in an accompanying article^[^ ^70^ ^]^ and briefly summarized here. (*S*)‐(+)‐ and (*R*)‐(−)‐thiofenchones were synthesized from the respective fenchone enantiomers with Lawesson's reagent (*Alfa Aesar*) in an *o*‐xylene solution at 155 °C. (*S*)‐(+)‐ and (*R*)‐(−)‐selenofenchone were synthesized from the respective fenchone enantiomers suspended in purified mesitylene using bis(1,5‐cyclooctanediylboryl) monoselenide at 120 °C. The latter was prepared in situ from 9‐borabicyclo[3.3.1]nonane‐dimer (*Merck*) at elevated temperatures above 150 °C according to literature procedures.^[^ ^71^, ^72^ ^]^

### 2.2 Nanosecond 2 + 1 REMPI Spectroscopy

Linearly polarized ns laser pulses were intersected with a pulsed molecular beam in the interaction region of a velocity map imaging (VMI) spectrometer,^[^ ^73^ ^]^ which was operated as a time‐of‐flight mass spectrometer. Here, we integrated all detected masses to obtain the total ion yield. The cold molecular beam was created by coexpanding helium and the respective molecular sample, which was heated to about 70 °C.^[^ ^74^, ^75^ ^]^ For fenchone, we used a frequency‐doubled commercial dye laser to produce wavelengths between 412 and 417 nm (*LIOP‐TEC*, pumped by the second harmonic of a Nd:YAG laser at 532 nm, dye: Styryl 9). For thiofenchone and selenofenchone, we used the fundamental of a different commercial dye laser (*Sirah*, pumped by the third harmonic of a Nd:YAG laser at 355 nm) to produce wavelengths from 441 nm to 446 nm (dye: Coumarin 120) and from 463 nm to 468 nm (dye: Coumarin 47) without frequency doubling. The laser pulse energy was in the range of 0.5 mJ to 1 mJ and the bandwidth below 0.2 nm. A plano‐convex lens of 300 mm focal length was used to focus the pulses in the interaction region of the VMI spectrometer. The repetition rate of the laser was 10 Hz.

The 3*p* Rydberg states of fenchone (see Figure 3B) were characterized with a different experimental setup. A frequency‐doubled commercial dye laser was used to produce laser wavelengths between 383 and 395 nm (*Sirah*, *Cobra‐Stretch*, pumped by the second harmonic of a Nd:YAG laser at 532 nm, dye: Styryl 11). The laser pulse energy was around 1.1 mJ at a repetition rate of 20 Hz and a bandwidth below 0.5 nm. The pulses were linearly or circularly polarized and focused into a VMI spectrometer operated in time‐of‐flight mode, similar to the setup described above, but with a moderately cold continuous molecular beam of fenchone seeded in helium.

### 2.3 Multiphoton Photoelectron Spectroscopy

Starting from femtosecond pulses from a 3 kHz repetition rate titanium‐sapphire amplifier, we generated ≈0.4 ps long pulses with a spectral width of ≈0.6 nm by second harmonic generation using a 5 mm thick *β*‐BBO crystal. By changing the phase‐matching angle, we tuned the wavelength of the second harmonic from 375 to 411 nm (6–9 μJ pulse energy), as characterized with a commercial grating spectrometer (*Avantes ULS3648*). A plano‐convex lens with 250 mm focal length was used to focus the pulses in the interaction region of a VMI spectrometer.^[^ ^73^ ^]^ The molecular sample was introduced by backfilling the vacuum chamber to a pressure of about 4 × 10^−6^ mbar. From the recorded 2D photoelectron images, the cylindrically symmetric 3D photoelectron momentum distributions were reconstructed by a rBasex algorithm implemented in PyAbel.^[^ ^76^ ^]^ From the momenta, the photoelectron energies were calculated. The energy axis was calibrated with photoelectrons derived from ionizing xenon atoms with the third harmonic of a Q‐switched Nd:YAG ns laser at 355 nm.

### 2.4 Single‐Photon VUV Absorption

A home‐built absorption set‐up has been used to measure single‐photon gas‐phase VUV absorption spectra. A deuterium lamp (*Hamamatsu L11798*) served as light source, which produced a continuous spectrum from 115 to 400 nm. The spectrum has been dispersed by a commercial normal‐incidence grating spectrometer with 1 m focal length in Rowland geometry (*McPherson Type 225* with 1200 lines mm^−1^). The dispersed light was focused on a photodiode behind the exit slit. A motorized grating rotation/translation system was used to scan the desired spectral range. A 2 cm path length target cell mounted between the deuterium lamp and the spectrometer's entrance slit was equipped with magnesium fluoride windows. Liquid samples were filled into the target cell at room temperature, which was briefly evacuated to remove residual gas before being heated up to 60 °C for fenchone and to 40 °C for thiofenchone and selenofenchone. A reference measurement of the lamp spectrum without sample was used to calculate the relative absorption via the Beer–Lambert law. The required high vapor pressure of the samples inside the cell led to the rapid formation of deposits on the windows and, consequently, short time frames for each measurement. Thus, 15 spectra were measured for each molecule, averaged and smoothed in segments, and stitched together. The resulting spectral resolution in the experiments was estimated to be about 0.1 eV. The wavelength axis has been calibrated using the absorption spectrum of nitrogen^[^ ^77^ ^]^ and the emission spectrum of a mercury arc lamp.

### 2.5 Femtosecond 2 + 1 REMPI‐PECD

The optical layout of the femtosecond PECD experiment is shown in Figure 1A. Laser pulses from a 3 kHz repetition rate titanium‐sapphire amplifier centered at 785 nm (*Femtopower HE*) with a pulse duration of below 25 fs and a pulse energy of 0.4 mJ were used to pump a commercial optical parametric amplifier (OPA) with a subsequent frequency conversion stage (*LightConversion TOPAS prime* with *NirUVis* extension), which produced output pulses centered at 376 nm with a spectral width of 8.3 nm. A thin nanowire grid broadband polarizer (*Quantum Design* 300–1000 nm) was used to ensure that the polarization of linearly polarized light was perpendicular to the acceleration direction of the spectrometer (see Figure 1A). Circularly polarized light was generated by an achromatic quarter‐wave plate (*B. Halle*, 300–470 nm). The circularity of the light was in all cases well above 99%, as measured via the Stokes parameter |S3|.^[^ ^78^ ^]^ A plano‐convex fused silica lens with 250 mm focal length (*Eksma Optics*) was used to focus the UV laser pulses with 3 μJ energy into the interaction region of a VMI spectrometer,^[^ ^73^ ^]^ which can be operated for ions or electrons.

A prism compressor consisting of two UV fused silica prisms was used to compensate for chirp, and bandwidth‐limited pulses were achieved in the interaction region by maximizing the multiphoton ionization signal of xenon. Assuming transform‐limited compression, the pulse duration was estimated to be around 25 fs (FWHM). For the measured Gaussian beam spot radius of 48 μm, we calculated a peak intensity of 3×10^12^ Wcm^−2^. The Keldysh parameter^[^ ^79^ ^]^ *γ* was estimated to be around 10, confirming the multiphoton ionization regime. The sample supply lines between the reservoir and the nozzle were heated to about 60 °C. The nozzle (*Agarscientific*, diameter: 100 μm) was heated to about 90 °C. The sample reservoir outside the vacuum chamber was heated to about 30 °C for fenchone, while for thiofenchone and selenofenchone, it was heated to about 50 °C. Conditions for a molecular beam were not met; hence, the nozzle was mainly used to maintain a pressure gradient between the supply line and the vacuum chamber. The experiment was operated with the chamber filled to a pressure of 3×10^−6^ mbar. Before the PECD experiment, we measured the mass spectra to ensure we had only the desired molecular substance (see Appendix Section A1).

Photoionization occurred between the repeller and extractor electrodes of the VMI spectrometer (see Figure 1A). Photoelectron momentum distributions were recorded on a position‐sensitive detector, a microchannel plate (MCP) coupled to a phosphor screen imaged by a CCD camera (*Lumenera Lw165m*). We accumulated events over 975,000 laser shots (130 ms exposure time, 2500 images) for each of the left‐circular (LCP), right‐circular (RCP), and linear (LIN) polarizations. After every 500 images, the polarization was switched to minimize artifacts due to long‐term experimental drifts. PECD images were derived by subtracting RCP from LCP photoelectron angular distributions. The resulting 2D distributions were Abel‐inverted to reconstruct the 3D momentum distributions using the rBasex algorithm implemented in PyAbel.^[^ ^76^ ^]^ The Abel inversion is performed using Legendre expansions of the electron momentum distributions. The retrieved Legendre coefficients *c*_*l*_ allow us to calculate the LPECD^[^ ^18^ ^]^

(1) $$\text{LPECD}=\frac{1}{c_{0}}\left(2c_{1}-\frac{1}{2}c_{3}+\frac{1}{4}c_{5}\right)$$

To account for the different enantiomeric excesses of the samples, LPECD values for (*R*)‐(−)‐fenchone were multiplied by the factor 1/0.84. We proceeded likewise for the (*R*) enantiomers of thiofenchone and selenofenchone to check if the enantiomeric excess is conserved during synthesis.

## 3 Computational Methodology

The TDDFT and CC2 calculations presented in this article were carried out with the TURBOMOLE (V7.6.0) program package.^[^ ^80^ ^]^ Electronic ground‐state structures for all chalcogenofenchones were optimized for the energy gradient using DFT with the long‐range corrected hybrid functional CAM‐B3LYP.^[^ ^81^, ^82^ ^]^ The resulting structures were confirmed to be minima on the respective potential energy surfaces by a harmonic vibrational frequency analysis. In all systems, the aug‐cc‐pVTZ basis set was chosen for C and H. For an improved description of the Rydberg states, quadruply augmented basis sets were used for the chalcogen centers. Specifically, we used q‐aug‐cc‐pVTZ for O, q‐aug‐cc‐pV(T + d)Z for S, and q‐aug‐cc‐pVTZ‐PP for Se, Te, and Po as the basis sets. The basis sets with the q‐aug moniker provide 4 (3 for the PP basis sets) diffuse functions also for the high *l*‐quantum numbers, providing ample freedom also for the higher‐lying states up to *f*‐type Rydberg states, with likely only the very high *g*‐like states affected in terms of quality. Additionally, the calculations were repeated with the diffuse functions of these basis sets anchored to the center of mass of the systems, which was confirmed not to impact the results to any significant degree. Relativistic small‐core pseudopotentials were used on Se, Te, and Po.

All Turbomole calculations applied the multipole‐accelerated resolution‐of‐identity (MARI‐J) approximation for the Coulomb energy contribution using the corresponding RI aug‐cc‐pVTZ basis sets (jbas) for all the atoms. The m4 integration grid was used for numerical integration to determine exchange–correlation contributions. The threshold for the self‐consistent field (SCF) energy convergence was set at 10^−9^ *E*_h_. Structure optimizations were performed until the norm of the energy gradient fell below $5\times 10^{-4}E_{h}a_{0}^{-1}$ and the displacements were smaller than 5 × 10^−4^ *a*_0_. The norm of the analytic gradients of the energy with respect to displacements of the nuclei was below $5\times 10^{-3}E_{h}a_{0}^{-1}$, and the estimated energy change between two optimization steps was below 5×10^−6^ *E*_h_. Single‐point calculations for each of the cations were performed at the same level of theory—using the equilibrium structure of the neutral electronic ground state—to obtain the vertical ionization energies.

The ground‐state energy minimum structure was used to calculate vertical excitation energies and oscillator strengths using time‐dependent DFT (TDDFT), as implemented in the Turbomole package. At least 20 low‐lying electronically excited states were calculated for each system, according to the experimental energy ranges. Furthermore, two‐photon absorption (TPA) cross sections for linear and circular polarized light were obtained at default settings using TDDFT.

To evaluate the significance of the TDDFT results, we compare them to the wave function‐based second‐order approximate CC method CC2,^[^ ^83^ ^]^ using the implementation in Turbomole. CC2 is expected to offer energies at the quality of second‐order Møller–Plesset perturbation theory (MP2) quality while giving access to excited state energies and transition moments. The excited‐state energies converged to an accuracy of 10^−7^ *E*_h_, and the vector function converged to 10^−4^ *E*_h_ while using a resolution of the identity parametrization for both the Coulomb and exchange integrals (RI‐JK). To provide a further measure of the quality of the methods, we also used EOM‐CCSD using MolPro.^[^ ^84^ ^]^ Since the comparison was focused on trends in Rydberg and valence excitation, we included the first 20 states for fenchone, thiofenchone, and selenofenchone.

To assign the *m*‐quantum number related character of *p* (*p*_*x*_, *p*_*y*_, *p*_*z*_) and *d* (*d*_*xy*_, *d*_*xz*_, *d*_*yz*_, $d_{z^{2}}$, $d_{x^{2}-y^{2}}$) and higher *l*‐quantum number Rydberg orbitals, we computed natural transition orbitals (NTOs) and visualized them for assignment. NTOs are obtained by using the singular vectors from a singular value decomposition of the transition density *P* ^*ia*^ between states *i* and *a* to transform the orbitals of the system.

(2) $$P^{ia}=UDV^{†}$$

where *D* is a diagonal matrix containing the singular values that determine the contribution of each NTO for the transition. The left singular vectors *U* are then related to the *hole* left by the excited electron from the initial state *i*, corresponding approximately to an occupied orbital determined by the orbital mixing coefficients *U*. Equivalently, the right singular vectors *V* describe the final state *a* of the excited *particle*, corresponding approximately to unoccupied molecular orbitals of valence‐ or Rydberg‐type.

**Figure 2:**
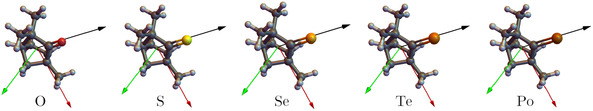
Optimized molecular structures of the (1*R*, 4*S*)‐chalcogenofenchones as identified by the atomic symbols of the chalcogenes. The green, red, and black arrows show the chosen *x*, *y*, and *z*‐axes, respectively.

A coordinate system based on the local symmetry of the formaldehyde‐like group containing the chalcogen atom was introduced to identify the *m*‐quantum number related character of the orbitals. Choosing the C=X bond (with X either O, S, Se, Te or Po) as the *z*‐axis, the plane encompassing the next‐neighboring C atoms is chosen to contain the *y*‐axis, with the *x*‐axis normal to this plane (cf. **Figure** **2**). It should be noted that the Rydberg‐like NTOs are not always aligned with this coordinate system perfectly and may also choose another axis than *z* as their principal axis. We chose to keep the coordinate system fixed for all molecules and states, and instead change the basis according to the shape and orientation of the NTOs. That means, for example, that if a *d*_*zz*_‐like orbital is rotated so that its principal axis is the *x*‐axis in our choice of coordinates, we denote it as *d*_*xx*_, with the other orbitals for the same *l*‐quantum number also adjusted to the new principal axis, e.g., *d*_*xx*−*yy*_ → *d*_*yy*−*zz*_, so that the choice is consistent.

## 4 Assignment of Excited States Through Spectroscopy

In this section, we present our findings of the spectroscopic experiments on fenchone, thiofenchone, and selenofenchone. For each molecule, the discussion is organized in the following way: As a first step, we assign the lowest lying Rydberg state of *s* symmetry using ns 2 + 1 REMPI spectra. With this, we determine the adiabatic ionization energy *I*_*P*_ using, in addition, multiphoton photoelectron spectra at different excitation wavelengths ranging from 375 to 411 nm. Then, we assign further electronic states using the adiabatic *I*_*P*_, multiphoton photoelectron spectra, and single‐photon VUV absorption spectra together with ab initio quantum chemical calculation.

### 4.1 Fenchone

**Figure 3:**
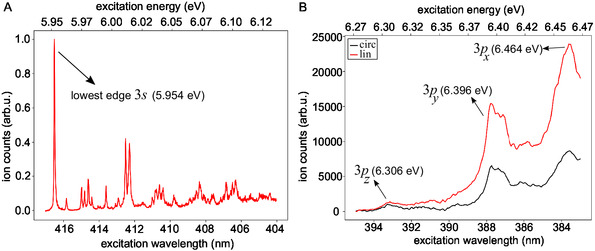
Nanosecond 2 + 1 REMPI spectrum of fenchone covering A) the lower vibrational levels of the 3*s* Rydberg state measured with linearly polarized laser pulses at high spectral resolution and (B) the 3*p* Rydberg states measured with linear (red) and circular (black) laser polarizations. The spectrum in (A) was normalized to its maximum. In B), no normalization was performed, but the acquisition time for both polarizations was the same.

**Figure** **3**A shows a highly‐resolved ns 2 + 1 REMPI spectrum of fenchone. The intense peak at 416.5 nm is the onset of the spectrum and indicates the threshold for reaching the 3*s* Rydberg state. Considering the two‐photon character for reaching this resonance, we obtain (5.954 ± 0.002) eV as the energy difference between the 3*s* Rydberg state and the electronic ground state, which agrees with earlier experiments.^[^ ^50^, ^63^, ^64^, ^85^ ^]^ At 6.300 eV, the TDDFT model overestimates the measured value, while CC2 underestimates it at 5.645 eV. As the calculations often yield more precise relative energies between different excited states than the absolute energies, **Table** **1** and **2** also contain columns of energies $E_{\text{calc}}^{*}$ that are shifted by the difference of the respective calculated 3*s* energy to the measured value. In addition to the intense peak attributed to the vibrational ground state of the 3*s*, the spectrum displays a pronounced progression—the partially resolved vibrational structure of the 3*s* electronic state.

**Table 1:** TDDFT results for fenchone: vertical electronic singlet excitation energies *E*_calc_, calculated energies $E_{\text{calc}}^{*}$ shifted such that the 3*s* energy matches the measured value, experimental state energies *E*_exp_, two‐photon excitation cross sections for linearly (circularly) polarized light *σ*_lin_ (*σ*_circ_) and oscillator strengths *f*. Parentheses mark a tentative assignment; italics in $E_{\text{calc}}^{*}$ mark states with significant valence character.

Electronic transition	*E*_calc_ [eV]	*E*_calc_* [eV]	*E*_exp_ [eV]	*σ*_lin_ [10^−50^ cm^4^ s]	*σ*_circ_ [10^−50^ cm^4^ s]	*f*
$n_{y}\to \pi_{x}^{*}$	4.336	*3.990*		0.0000	0.0000	0.0000
*n*_*y*_ → 3*s*	6.300	5.954	5.954	0.0108	0.0161	0.0004
*n*_*y*_ → 3*p*_*z*_	6.738	6.392	6.306	0.0032	0.0047	0.0249
*n*_*y*_ → 3*p*_*y*_	6.770	6.424	6.396	0.0310	0.0124	0.0151
*n*_*y*_ → 3*p*_*x*_	6.816	6.4700	6.464	0.0112	0.0040	0.0040
*n*_*y*_ → 3*d*_*xx*_	7.226	6.880	6.85	0.0120	0.0165	0.0054
*n*_*y*_ → 3*d*_*yz*_	7.338	6.992		0.0553	0.0074	0.0075
*n*_*y*_ → 3*d*_*xy*_	7.351	7.005		0.0185	0.0122	0.0006
*n*_*y*_ → 3*d*_*yy*−*zz*_	7.380	7.034	7.04	0.0108	0.0044	0.0155
*n*_*y*_ → 3*d*_*xz*_	7.409	7.063		0.0070	0.0069	0.0010
*n*_*y*_ → 4*s*	7.454	7.108		0.0020	0.0017	0.0007
$\sigma_{1}\to \pi_{x}^{*}$	7.578	7.232		0.0019	0.0008	0.0173
*n*_*y*_ → 4*p*_*x*_	7.601	7.255	7.46	0.0003	0.0002	0.0006
*n*_*y*_ → 4*p*_*z*_	7.613	7.267		0.0001	0.0001	0.0042
*n*_*y*_ → 4*p*_*y*_	7.620	7.274		0.0005	0.0002	0.0018
*n*_*y*_ → 5*p*_*x*_	7.855	7.509		0.0013	0.0019	0.0002
*n*_*y*_ → 5*p*_*y*_ + 5*p*_(*z*)_	7.878	7.532		0.0004	0.0028	0.0054
*n*_*y*_ → 5*p*_*z*_ − 5*p*_(*y*)_	7.912	7.566		0.0007	0.0004	0.0017
*n*_*y*_ → 4*d*_(*yz*)_	7.917	7.571		0.0029	0.0043	0.0171
*n*_*y*_ → 4*d*_(*xx*−*zz*)_	7.934	7.588		0.0010	0.0014	0.0002
*n*_*y*_ → 4*d*_(*yy*)_	7.952	7.606		0.0028	0.0032	0.0043
*n*_*y*_ → 4*d*_(*xy*)_	8.018	7.672		0.0035	0.0040	0.0061
*n*_*y*_ → 4*d*_(*xz*)_	8.027	7.681		0.0020	0.0018	0.0017
*σ*_−1_ → 3*s*	8.086	7.740		0.0152	0.0095	0.0035
*n*_*y*_ → 4*f*_(*yyy*−3*xxy*)_	8.116	7.77		0.0123	0.0174	0.0060
*n*_*y*_ → 4*f*_*xxy*_( + 6*s*)	8.160	7.814		0.0231	0.0212	0.0003
*σ*_−3_ → 5*s*	8.176	7.830		0.0009	0.0009	0.0138

**Table 2:** CC2 results for fenchone: vertical electronic singlet excitation energies *E*_calc_, calculated energies $E_{\text{calc}}^{*}$ shifted such that the 3*s* energy matches the measured value, experimental state energies *E*_exp_, and oscillator strengths *f*. Parentheses mark a tentative assignment; italics in $E_{\text{calc}}^{*}$ mark states with significant valence character.

CC2 fenchone				
Electronic transitions	*E*_calc_ [eV]	*E*_calc_* [eV]	*E*_exp_ [eV]	*f*
$n_{y}\to \pi_{x}^{*}$	4.356	*4.665*		0.0000
*n*_*y*_ → 3*s*	5.645	5.954	5.954	0.0032
*n*_*y*_ → 3*p*_*z*_	5.980	6.289	6.306	0.0137
*n*_*y*_ → 3*p*_*y*_	6.024	6.333	6.396	0.0127
*n*_*y*_ → 3*p*_*x*_	6.075	6.384	6.464	0.0020
*n*_*y*_ → 3*d*_*xx*_	6.483	6.792	6.85	0.0058
*n*_*y*_ → 3*d*_*xy*_	6.561	6.870		0.0005
*n*_*y*_ → 3*d*_*yz*_	6.565	6.874		0.0052
*n*_*y*_ → 3*d*_*yy*−*zz*_	6.590	6.899	7.04	0.0103
*n*_*y*_ → 3*d*_*xz*_	6.609	6.918		0.0028
*n*_*y*_ → 4*s*	6.703	7.012		0.0009
*n*_*y*_ → 4*p*_*z*_	6.882	7.191		0.0055
*n*_*y*_ → 4*p*_*x*_ + 4*p*_*y*_	6.890	7.199	7.46	0.0030
*n*_*y*_ → 4*p*_*y*_ + 4*p*_*x*_	6.903	7.212		0.0051
*n*_*y*_ → 4*f*_*yzz*_ + 4*p*_*y*_	7.084	7.393		0.0023
*n*_*y*_ → 4*d*_*yz*_	7.116	7.425		0.0074
*n*_*y*_ → 4*d*_*xz*_	7.146	7.455		0.0011
*n*_*y*_ → 4*d*_(*yy*−*zz*)_	7.178	7.487		0.0091
*n*_*y*_ → 5*p*_*x*_	7.184	7.493		0.0020
*n*_*y*_ → 5*d*_*xz*_	7.207	7.516		0.0059

Figure 3B displays a ns 2 + 1 REMPI spectrum of the 3*p* Rydberg states recorded with linearly and circularly polarized light. From this measurement, we obtained (6.306 ± 0.007) eV for the weak signal of the lowest‐energy 3*p* state. Following the suggestion of Powis et al.^[^ ^64^ ^]^ we assign this state as 3*p*_*z*_. It has a (0.352 ± 0.007) eV higher energy than the 3*s* state. This difference is nearly matched by the CC2 calculation at 0.352 eV but overestimated by the TDDFT model at 0.438 eV. The 3*p*_*z*_ state assignment is supported by the observation of a relatively high signal for circular polarization as compared to linear polarization, in agreement with existing calculations^[^ ^63^, ^64^ ^]^ and with our calculated two‐photon cross sections in Table 1. We must add that comparing the 2 + 1 REMPI signal strength with calculated two‐photon cross sections is only reasonable if the ionization step is saturated, i.e., the excited molecules are likely to be ionized. This has been verified at similar laser conditions by Singh et al.^[^ ^63^ ^]^

For the second 3*p* state (3*p*_*y*_), we measured an energy of (6.396 ± 0.007) eV, which agrees with the earlier work of Powis.^[^ ^64^ ^]^ The difference to the 3*s* energy is 0.442 eV, which is underestimated by 0.06 eV in the CC2 model and overestimated by 0.03 eV in the TDDFT. We suggest to assign the intense peak above 384 nm in the ns 2 + 1 REMPI spectrum (Figure 3B) to the third 3*p* state (3*p*_*x*_) with an energy of (6.464 ± 0.007) eV. This suggestion differs from the recent work of Powis and Singh,^[^ ^64^ ^]^ who locate the 3*p*_*x*_ band origin in the same broad peak as the 3*p*_*y*_ band origin. Our assignment is supported by the energy differences calculated by our TDDFT and CC2 models (see Table 1 and 2). Moreover, the observed lower circular‐to‐linear ratio of the 3*p*_*x*_ compared to the 3*p*_*y*_ peak fits well to our TDDFT results (see Table 1) and older calculations.^[^ ^62^, ^63^, ^85^ ^]^ In energies relative to the 3*s* state, the calculated values for the 3*p*_*y*_ and 3*p*_*x*_ states are again slightly overestimated by the TDDFT model and more significantly underestimated by the CC2 model.

**Figure 4:**
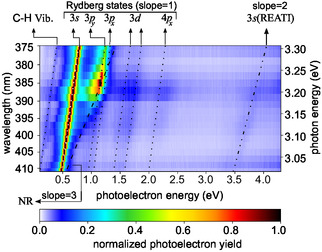
Multiphoton photoelectron spectra of fenchone. Each row in the figure represents a measurement at a different central wavelength. All measurements used a pulse duration of 0.4 ps and linear polarization. In each row, the signal is normalized to its maximum. Lines indicate linear scaling that can be attributed to different processes: 2 + 1 REMPI through the labeled Rydberg states yields a slope of one, and 2 + 2 REATI through Rydberg states a slope of two. In contrast, NR multiphoton ionization is associated with a slope of three. The corresponding second‐order Legendre coefficients are presented in Figure A2 in the Appendix.

**Figure A2:**
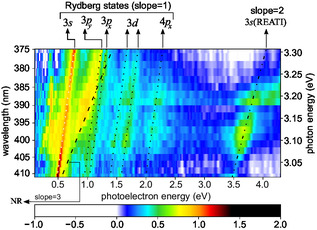
Ratio of second‐order Legendre coefficient *c*_2_ to the total signal *c*_0_ for the wavelength‐dependent multiphoton photoelectron spectra of fenchone presented in Figure 4.

For further insight, multiphoton photoelectron spectra were measured for 19 different central wavelengths ranging from 375 to 411 nm. **Figure** **4** presents all photoelectron spectra together such that it constitutes a map of the photoelectron yield as a function of the photoelectron energy and the energy of the laser photons. In addition to the map of the yield, we also consider the map of the second‐order Legendre coefficient of the photoelectron angular distributions (see **Figure** **A2** in the Appendix). These map‐like representations simplify the identification of different ionization mechanisms due to linear scaling of the photoelectron energies *E*_kin_ with the photon energy *hν*, represented by dotted lines in Figure 4. Most ionization mechanisms are described very well by lines with a slope of one. This matches previous observations^[^ ^36^, ^45^ ^]^ for a 2 + 1 REMPI process via a resonant Rydberg state (see Figure 1B). Due to the very similar potential energy surfaces of the neutral Rydberg states and the ionic ground state, the Franck–Condon factors for transitions with preserved vibrational quantum number dominate in the ionization step (Δ*v* = 0 propensity rule).^[^ ^36^, ^45^ ^]^ Under such conditions, the following equation for the photoelectron energy applies^[^ ^36^ ^]^

(3) $$E_{\text{kin}}(h\nu )=h\nu +E_{\text{Ryd}}-I_{P}$$

where *E*_Ryd_ denotes the Rydberg state energy and *I*_*P*_ the ionization energy.

In the following, we refer to the lines in Figure 4 and A2 by their photoelectron energy for the lowest photon energy at a wavelength of 411 nm (*hν* ≈ 3.01 eV). The line starting at 0.5 eV corresponds to the 3*s* state of fenchone. Fitting by Equation (3), with a 3*s* Rydberg state energy of $E_{\text{Ryd}}^{3s}=5.954\;\text{eV}$ extracted from the ns 2 + 1 REMPI spectrum, the adiabatic ionization energy was determined to be $I_{P}^{\text{fen}}=(8.48\pm 3)\;\text{eV}$ which is consistent with previous work.^[^ ^36^, ^63^ ^]^ Knowing the *I*_*P*_, we can fit the other lines with slope one in Figure 4 by Equation (3) to determine the energies *E*_Ryd_ of further Rydberg states, which we do in the following.

We tentatively assign the weak line below 0.5 eV to the 3*s* Rydberg state accompanied by a vibrational excitation in the C–H stretching band^[^ ^65^ ^]^ because the calculations do not hint at any Rydberg states below the 3*s* state. However, the energy difference between this line and the 3*s* line is around 0.36 eV, which matches the energy of the C–H stretch vibration.^[^ ^86^ ^]^ This notable exception to the Δ*v* = 0 propensity rule is much weaker than the 3*s* line resulting from Δ*v* = 0 and therefore indicates the strength of the propensity. Another trace of weak vibrational excitation during the ionization step can be found in the asymmetric shape of the 3*s* signal with a broader flank to lower photoelectron energies. These electrons could be assigned to Δ*v* > 0 for low‐energetic vibrational modes.

The photoelectron contributions around 1 eV belong to the 3*p* Rydberg states. The lowest 3*p* state (3*p*_*z*_), whose energy from the ns spectroscopy is (6.306 ± 0.007) eV, is not visible in the photoelectron spectra due to its low two‐photon cross section discussed earlier. The other two *p* states (3*p*_*y*_ and 3*p*_*x*_) are distinctly visible in the second Legendre coefficient (see Figure A2 in the Appendix). For the second 3*p* state (3*p*_*y*_), we obtain (6.41 ± 0.04) eV, which is in good agreement with the ns 2 + 1 REMPI assignment and also in accordance with earlier observations.^[^ ^36^, ^50^, ^63^, ^64^, ^85^ ^]^ For the third 3*p* state (3*p*_*x*_), we determine (6.50 ± 0.05) eV, which is also in agreement with the ns 2 + 1 REMPI result of (6.464 ± 0.007) eV. This is another confirmation of the 3*p* state assignment performed on the ns REMPI spectrum (Figure 3B).

Note that even at wavelengths longer than the two‐photon threshold, photoelectrons whose energies are consistent with Δ*v* = 0 ionization from the 3*p* states are detected, as seen in Figure 4 and in particular in Figure A2. We suspect that two mechanisms can explain these electrons: First, three photons promote an electron from the HOMO−1 orbital to one of the 3*p* orbitals. The HOMO−1 orbital is 1.8 eV more bound than the HOMO orbital.^[^ ^31^, ^51^ ^]^ Then, the molecule is in a doubly‐excited state of Rydberg character that has a potential energy surface similar to that of the excited molecular ion with a hole in the HOMO−1 orbital. Another photon can ionize the molecule to this ionic state without changing its vibrational quantum numbers (Δ*v* = 0 propensity). The other possible mechanism exploits that off‐resonant states are populated transiently during multiphoton transitions. This transient population vanishes at the end of the laser pulse, but the molecule can be ionized with a low probability during the laser pulse from the transiently populated 3*p* Rydberg states. The energy of the photoelectron will then carry the energy associated with these states.^[^ ^87^, ^88^ ^]^ Therefore, in both mechanisms, the ionization step takes place via a Rydberg state, and Equation (3) can be used to determine the state's energy.

Above the photoelectron energy of 1.2 eV, we can see three further lines of photoelectron signals with a slope of one. These feature low count rates because the corresponding higher‐lying Rydberg states cannot be reached by two photons at all photon energies. Therefore, these photoelectrons can be attributed to either 3 + 1 REMPI, including a hole in the HOMO−1 orbital, or to the ionization of transiently populated states. We find two photoelectron contributions around 1.5 eV which correspond to Rydberg energies of (6.85 ± 0.06) and (7.04 ± 0.06) eV. Around these and higher energies, the models predict a vast number of states, which makes a purely energetic assignment challenging. We therefore computed quantum defects $\delta =n-\sqrt{13.6\;\text{eV}/\left(I_{P}-E_{\text{Ryd}}\right)}$ ^[^ ^89^ ^]^ for the previously assigned states and the higher energy values. The quantum defect is usually similar for different Rydberg states of one atom or molecule with the same angular momentum *l* but different principal quantum numbers *n*. This behavior has, for example, been confirmed experimentally for the ketones acetone^[^ ^90^ ^]^ and methyl‐substituted cyclopentanones.^[^ ^91^ ^]^ Here, we will use this behavior to select states out of the many that are energetically possible according to our calculations.

Based on the energy difference to the 3*s* state, the lower state with *E*_Ryd_ = (6.85 ± 0.06) eV is likely a 3*d* state but could also be a 4*s* or 4*p* Rydberg state. Calculating *δ* for *n* = 4 yields a quantum defect distinct from those obtained for the 3*s* and 3*p* states. Therefore, we conclude that this state belongs to the 3*d* manifold. The corresponding quantum defect is smaller than those for *s* and *p* states, which matches the previously observed trend of decreasing *δ* with increasing *l*.^[^ ^89^, ^90^ ^]^ Based on the TDDFT energies, we prefer the assignment as 3*d*_*xx*_. Likewise, we conclude from the quantum defect calculation that the Rydberg state at 7.04 eV is 3*d* as well, where we prefer either 3*d*_*yy*−*zz*_ or 3*d*_*xz*_ (see Table 1). In the CC2 model, the relative energies of these states are about 0.15 eV smaller, but we still see this as a confirmation, considering the general tendency of CC2 to underestimate the energy differences between states.

We observe another weak contribution around 2 eV, which corresponds to a Rydberg energy of (7.46 ± 0.07) eV. By comparing the calculated quantum defect with those of the known Rydberg states, we prefer an assignment of this state as 4*p*, with 4*p*_*x*_ being the most likely candidate.

Additionally, Figure 4 and A2 contain two lines of photoelectron energies that do not have a slope of one as a function of the photon energy. First, there is a region of photoelectrons with energies approaching a line with a slope of three that starts slightly above the photoelectron energy of 0.5 eV. These electrons have a stronger signature in the second‐order Legendre coefficient *c*_2_ (see Figure A2) and correspond to the nonresonant (NR) three‐photon ionization, where the molecule is directly ionized without passing through an intermediate state. In this case, electrons should have a low probability for zero angular momentum, which explains the stronger signature in *c*_2_. The electrons do not reach the energy of 3*hν* − *I*_*P*_ that is represented in the figure because of vibrational excitation in the molecular ion. Second, we observe a line around 3.5 eV photoelectron energy, particularly pronounced in Figure A2. It has a slope of two, and its photoelectron energies are always one photon energy higher than those corresponding to the 2 + 1 REMPI process via the 3*s* Rydberg states. This implies that this high‐energy contribution belongs to a 2 + 2 resonance‐enhanced above‐threshold ionization (REATI) through the same state.^[^ ^92^, ^93^ ^]^

**Figure 5:**
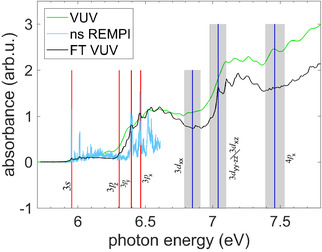
Single‐photon VUV absorption spectrum of gas‐phase fenchone measured with a deuterium lamp (green) in comparison with ns 2 + 1 REMPI (cyan)^[^ ^50^ ^]^ and FT VUV (black).^[^ ^63^ ^]^ Note that the absorbance scales are individually arbitrary for all spectra. Vertical red lines show the state energies from the ns 2 + 1 REMPI spectra, where the lines are thicker than the measured uncertainties. Blue vertical lines indicate the state energies from the multiphoton photoelectron spectra, with the uncertainties shaded in gray.

In **Figure** **5**, we present our single‐photon VUV absorption spectrum of fenchone (green line). Although its relative absorption increasingly deviates from the FT‐VUV spectrum of Singh et al. (black line),^[^ ^63^ ^]^ resonant structures are reproduced quite well. The deviation may be due to the ill‐defined absolute emission of the deuterium lamp. As the determination of the absolute magnitude of absorption, however, was not the target of the present investigation, rather than determining energies of resonances, we did not perform separate experiments to quantify the incoming photon flux. We mark the energy values of the 3*s* and 3*p* Rydberg states obtained from the current ns 2 + 1 REMPI spectra with red vertical lines. These energy values agree with both VUV spectra and a high‐resolution ns REMPI spectrum (cyan line).^[^ ^36^ ^]^ Furthermore, vertical lines in dark blue mark the energy values of the 3*d* and 4*p* states obtained from the multiphoton photoelectron spectra, with the errors shaded in grey. The 3*d* and 4*p* energies are consistent with the band origins in the single‐photon absorption spectra within the estimated errors (see Figure 5). Note that VUV absorption spectra feature vibrational progressions of the Rydberg states and possibly absorption into non‐Rydberg states. The overall shapes of both measured single‐photon VUV spectra fit reasonably well with the calculated oscillator strengths *f* of the TDDFT model (see Table 1). In Figure 5, the 3*s* Rydberg state has a lower signal than the 3*p* states; the same is true for the calculated *f* values. However, the calculated oscillator strengths of most of the 3*d* states are weaker than those of the 3*p* states, which only partially agrees with the measured VUV single‐photon spectrum.

### 4.2 Thiofenchone

**Figure 6:**
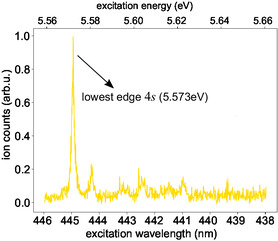
Nanosecond 2 + 1 REMPI spectrum of thiofenchone covering the lowest edge of the 4*s* Rydberg state measured with linearly polarized laser pulses.

**Figure** **6** depicts the ns 2 + 1 REMPI spectrum measured for thiofenchone. The intense peak at 444.9 nm is the onset of the spectrum and indicates the threshold for reaching the 4*s* Rydberg state. Considering the two‐photon character for reaching this resonance, we obtain (5.573 ± 0.003) eV as the energy difference between the 4*s* electronic state and the electronic ground state. This compares favorably with the value of 5.55 eV, which was measured at lower resolution in gas‐phase UV absorption.^[^ ^67^ ^]^ In addition, the measured 4*s* Rydberg state energy is in good agreement with the energy values obtained from the TDDFT (5.597 eV, see **Table** **3**) and CC2 (5.492 eV, see **Table** **4**) calculations. The smaller peaks at higher photon energies in the REMPI spectrum of Figure 6 are attributed to excited vibrational levels of the 4*s* electronic state.

**Table 3:** TDDFT results for thiofenchone: vertical electronic singlet excitation energies *E*_calc_, calculated energies $E_{\text{calc}}^{*}$ shifted such that the 4*s* energy matches the measured value, experimental state energies *E*_exp_, two‐photon excitation cross sections for linearly (circularly) polarized light *σ*_lin_ (*σ*_circ_) and oscillator strengths *f*. Parentheses mark a tentative assignment; italics in $E_{\text{calc}}^{*}$ mark states with significant valence character.

TDDFT thiofenchone						
Electronic transitions	*E*_calc_ [eV]	*E*_calc_* [eV]	*E*_exp_ [eV]	*σ*_lin_ [10^−50^ cm^4^ s]	*σ*_circ_ [10^−50^ cm^4^ s]	*f*
$n_{y}\to \pi_{x}^{*}$	2.635	*2.611*		0.0000	0.0000	0.0000
$\pi_{x}\to \pi_{x}^{*}$	5.517	*5.493*		0.0102	0.0009	0.2232
*n*_*y*_ → 4*s*	5.597	5.573	5.573	0.0182	0.0273	0.0248
$\sigma_{-2}\to \pi_{x}^{*}$	5.969	*5.945*		0.0006	0.0009	0.0145
*n*_*y*_ → 4*p*_*z*_	6.068	6.044	5.99	0.0037	0.0056	0.0123
*n*_*y*_ → 4*p*_*x*_	6.157	6.133	6.11	0.0075	0.0079	0.0002
*n*_*y*_ → 4*p*_*y*_	6.186	6.162		0.0100	0.0020	0.0038
*n*_*y*_ → 3*d*_*xx*−*zz*_	6.461	6.437	6.46	0.0157	0.0234	0.0012
$\pi_{y}\to \pi_{x}^{*}$	6.481	*6.457*		0.0016	0.0022	0.0009
*n*_*y*_ → 3*d*_*yy*_	6.648	6.624	6.62	0.0029	0.0034	0.0007
*n*_*y*_ → 3*d*_*yz*_	6.666	6.642		0.0947	0.0145	0.0020
*n*_*y*_ → 3*d*_(*xy*)_	6.678	6.654		0.0253	0.0114	0.0019
*n*_*y*_ → 3*d*_*xz*_	6.725	6.701		0.0071	0.0106	0.0004
*n*_*y*_ → 5*s*	6.771	6.747		0.0021	0.0018	0.0041
$\sigma_{-5}\to \pi^{x}$	6.803	*6.779*		0.0100	0.0120	0.0139
*n*_*y*_ → 5*p*_*z*_	6.893	6.869		0.0004	0.0003	0.0003
*n*_*y*_ → 5*p*_*x*_	6.909	6.885		0.0007	0.0009	0.0000
*n*_*y*_ → 5*p*_*y*_	6.928	6.904		0.0042	0.0013	0.0021
$\sigma_{-4}\to \pi_{x}^{*}$	6.949	*6.925*		0.0063	0.0027	0.0027
*n*_*y*_ → 6*p*_(*z*)_ (55.59%), $\sigma_{-3}\to \pi_{x}^{*}$ (43.9%)	7.014	*6.990*		0.0037	0.0044	0.0012
$\sigma_{-3}\to \pi_{x}^{*}$ (54.72%), *n*_*y*_ → 6*p*_*z*_ (44.61%)	7.022	*6.998*		0.0023	0.0026	0.0166
*n*_*y*_ → 6*p*_*x*_	7.135	7.111		0.0011	0.0017	0.0005
*n*_*y*_ → 6*p*_*y*_	7.156	7.132		0.0080	0.0018	0.0018
*n*_*y*_ → 4*d*_(*yz*)_ + *p*_*y*_	7.191	7.167		0.0012	0.0005	0.0056
*n*_*y*_ → 4*d*_*yy*−*zz*_	7.206	7.182	(7.14)	0.0019	0.0015	0.0033
*n*_*y*_ → 4*d*_*xy*_	7.211	7.187		0.0018	0.0008	0.0017
$\pi_{x}\to 6s$	7.236	7.212		0.0080	0.0105	0.0494

**Table 4:** CC2 results for thiofenchone: vertical electronic singlet excitation energies *E*_calc_, calculated energies $E_{\text{calc}}^{*}$ shifted such that the 4*s* energy matches the measured value, experimental state energies *E*_exp_, and oscillator strengths *f*. Parentheses mark a tentative assignment; italics in $E_{\text{calc}}^{*}$ mark states with significant valence character.

CC2 thiofenchone				
Electronic transitions	*E*_calc_ [eV]	*E*_calc_* [eV]	*E*_exp_ [eV]	*f*
$n_{y}\to \pi_{x}^{*}$	2.645	*2.726*		0.0000
*n*_*y*_ → 4*s*	5.492	5.573	5.573	0.0129
$\pi_{x}\to \pi_{x}^{*}$	5.624	*5.705*		0.2342
*n*_*y*_ → 4*p*_*z*_	5.826	5.907	5.99	0.0226
*n*_*y*_ → 4*p*_*x*_ + 4*p*_*y*_	5.958	6.039	6.11	0.0014
*n*_*y*_ → 4*p*_*y*_ + 4*p*_*x*_	5.976	6.057		0.0041
$\sigma_{1}\to \pi_{x}^{*}$	6.081	*6.162*		0.0227
*n*_*y*_ → 3*d*_*xx*−*zz*_	6.282	6.363	6.46	0.0009
*n*_*y*_ → 3*d*_*yz*_	6.415	6.496		0.0008
*n*_*y*_ → 3*d*_*yy*_	6.427	6.508	6.62	0.0027
*n*_*y*_ → 3*d*_*xz*_	6.441	6.522		0.0009
*n*_*y*_ → 3*d*_*xy*_	6.480	6.561		0.0003
*n*_*y*_ → 5*s*	6.574	6.655		0.0051
$\pi_{y}\to \pi_{x}^{*}$	6.671	*6.752*		0.0015
*n*_*y*_ → 5*p*_*z*_	6.761	6.842		0.0039
*n*_*y*_ → 5*p*_*x*_	6.786	6.867		0.0003
*n*_*y*_ → 5*p*_*y*_	6.800	6.881		0.0042
$\pi_{3}\to \pi_{x}^{*}$ (68.39%), *n*_*y*_ → 6*s* (31.14%)	6.882	*6.963*		0.0065
*n*_*y*_ → 6*s* (53.58%), $\pi_{3}\to \pi_{x}^{*}$ (46.%)	6.886	*6.967*		0.0178
*n*_*y*_ → 4*d*_*xx*−*zz*_	6.961	7.042	(7.14)	0.0033

**Figure 7:**
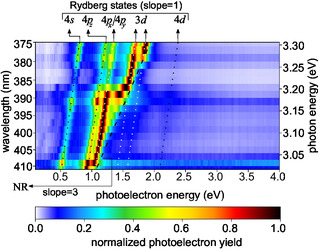
Multiphoton photoelectron spectra of thiofenchone. Each row in the figure represents a measurement at a different central wavelength. All measurements used a pulse duration of 0.4 ps and linear polarization. In each row, the signal is normalized to its maximum. Lines indicate linear scaling that can be attributed to different processes: 2 + 1 REMPI through the labeled Rydberg states yields a slope of one, while NR multiphoton ionization is associated with a slope of three. The corresponding second Legendre coefficients are presented in Figure A3 in the Appendix.

To determine the adiabatic *I*_*P*_ and to identify more states, we measured multiphoton photoelectron spectra for 19 different central wavelengths ranging from 375 to 411 nm, which is shown in **Figure** **7**. In analogy to the case of fenchone (Section 4.1), we identify intense lines with a slope of one as associated with Rydberg states via 2 + 1 REMPI. The line originating at 0.5 eV corresponds to the 4*s* Rydberg state of thiofenchone. Fit by Equation (3), using the 4*s* Rydberg energy from ns REMPI (5.573 eV), results in an adiabatic ionization energy $I_{P}^{\text{thi}o}=(8.07\pm 0.03)\;\text{eV}$ which is in close agreement with the vertical *I*_*P*_ of 8.1 eV measured by Sandström et al.^[^ ^66^ ^]^ and our TDDFT value of *I*_*P*_ = 8.15 eV.

**Figure A3:**
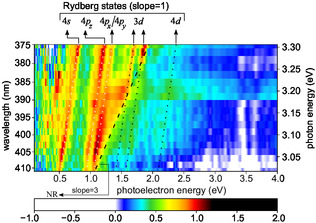
Ratio of second‐order Legendre coefficient *c*_2_ to the total signal *c*_0_ for the wavelength‐dependent multiphoton photoelectron spectra of thiofenchone presented in Figure 7.

With the knowledge of *I*_*P*_, we can use fits by Equation (3) to the other lines with a slope of one in Figure 7 and **A3** to determine the energies *E*_Ryd_ of further Rydberg states of thiofenchone. We attribute the two lines originating near 1 eV to 4*p* states located at (5.99 ± 0.05) and (6.11 ± 0.05) eV, respectively. These Rydberg states have also been observed by Falk and Steer,^[^ ^67^ ^]^ who identified them as 4*p*_*z*_ and 4*p*_*y*_, respectively. Further evidence for the assignment of the 4*p*_*z*_ state at (5.99 ± 0.05) eV is based on our calculations: While the energy difference to the 4*s* state from the TDDFT calculation is near the upper limit of the experimental error, the CC2 model yields a lower energy difference of 0.334 eV, which is close to the experimentally determined error range. The state at (6.11 ± 0.05) eV on the other hand may be attributed to either 4*p*_*y*_ or 4*p*_*x*_, which in the TDDFT calculation have a separation of just 0.03 eV—below our experimental resolution. Note that the CC2 model identifies the states as being of mixed (4*p*_*x*_ + 4*p*_*y*_) character. The calculated two‐photon excitation cross section from the TDDFT model for linearly polarized light is smaller for the 4*p*_*z*_ state than for the 4*p*_*x*_ and 4*p*_*y*_ states, which is in agreement with our observation of the line strength in Figure 7.

Figure 7 contains two lines originating around 1.5 eV, which show a threshold behavior near 388 and 380 nm, respectively. The energies of the corresponding states are determined to be (6.46 ± 0.06) and (6.62 ± 0.06) eV. Comparison of the energy separation to the 4*s* state with the TDDFT calculation (Table 3) suggests that the (6.46 ± 0.06) eV state is the lowest‐energy 3*d* state, 3*d*_*xx*−*zz*_. Considering the tendency of the CC2 model to underestimate the energy differences of the states, this assignment is supported by the CC2 model, too. Similarly, the TDDFT calculation suggests that the (6.62 ± 0.06) eV state is 3*d*_*yz*_ or/and 3*d*_*yy*_. Of these, the 3*d*_*yz*_ is much more likely to be populated in two‐photon excitation due to the higher cross section. The assignments of the two states at (6.46 ± 0.06) and (6.62 ± 0.06) eV as 3*d* states is supported by the calculated quantum defects, but the assignment of *m*‐states has to be considered as tentative. The multiphoton photoelectron spectra also show a weak line originating at around 2.1 eV, which can be attributed to either 3 + 1 REMPI, including a hole in the HOMO−1 orbital or to the ionization of transiently populated NR states. We obtain (7.14 ± 0.09) eV for this state, which, according to our quantum defect calculations is most likely a 4*d* state, but could also be a 5*p* state, which are also energetically feasible in both models. It was not possible to unambiguously assign this state to a specific sub‐state of the 4*d* or 5*p* manifolds.

In addition to the lines associated with Rydberg states, Figure 7 contains some signal at energies below the 4*s* Rydberg states. This signal is weak and does not follow a line. Similar traces can be seen in the second‐order Legendre coefficient (see **Figure** **A3**). These photoelectrons could be attributed to the ($\pi_{x}\to \pi_{x}^{*}$) valence state, which overlaps with the 4*s*, as we will see in the next paragraph.

**Figure 8:**
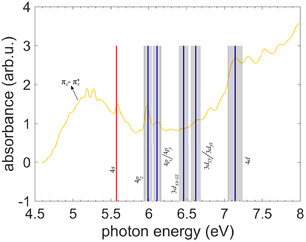
Single‐photon VUV absorption spectrum of gas‐phase thiofenchone measured with a deuterium lamp. The vertical red line marks the 4*s* state energy from the ns 2 + 1 REMPI spectra, where the line is thicker than the measured uncertainty. Blue vertical lines indicate the state energies from the multiphoton photoelectron spectra, with the uncertainties shaded in gray.

In **Figure** **8**, the Rydberg state energies of thiofenchone discussed above are compared with the measured single‐photon VUV gas‐phase absorption spectrum. Matching absorption peaks for the 4*s* and 4*p* states are found, while the higher‐lying Rydberg states are difficult to allocate because possible peaks are indiscernible from the noise (see Section 2.4). In addition to peaks attributed to Rydberg states, the VUV spectrum exhibits a broad feature starting at 4.6 eV and peaking around 5.2 eV, which is assigned to the ($\pi_{x}\to \pi_{x}^{*}$) state, which has valence character. For this state, both theories yield vertical transition energies, which are significantly higher than the measured peak value (TDDFT: 5.517 eV and CC2: 5.624 eV, i.e., even energetically above the 4*s* Rydberg state). This pronounced deviation may indicate that a detailed vibronic simulation of this broad ($\pi_{x}\to \pi_{x}^{*}$) transition profile would be required to establish the relation between peak maximum and vertical transition energy. Our measured single‐photon absorption spectrum reproduces most of the features of the earlier spectrum by Falk and Steer,^[^ ^67^ ^]^ but extends to higher energies. A notable difference is a small modulation on top of the ($\pi_{x}\to \pi_{x}^{*}$) absorption band around 5.2 eV, which, however, is attributed to experimental noise. In general, the oscillator strengths *f* calculated with TDDFT (see Table 3) for the ($\pi_{x}\to \pi_{x}^{*}$), 4*s*, and 4*p* states are reproduced in the general shape of the UV spectrum, but the model does not find a single state with large *f* that agrees with the strong increase in absorbance above 6.8 eV. This increase is likely due to the multitude of states in this energy region. At least the 4*d* state identified from the multiphoton photoelectron spectra is located there.

### 4.3 Selenofenchone

**Figure 9:**
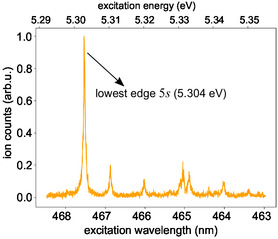
Nanosecond 2 + 1 REMPI spectrum of selenofenchone covering the lowest edge of the 5*s* Rydberg state measured with linearly polarized laser pulses.

**Figure** **9** shows a well‐resolved ns 2 + 1 REMPI spectrum of selenofenchone. The intense peak at 467.5 nm is the onset of the spectrum and indicates the threshold for reaching the 5*s* Rydberg state. Considering the two‐photon character for reaching this resonance, we obtain (5.304 ± 0.003) eV as the energy difference between the 5*s* Rydberg state and the electronic ground state, which agrees with the calculated TDDFT (**Table** **5**) and CC2 energy values (**Table** **6**). The smaller peaks at higher photon energies in the REMPI spectrum of Figure 9 are attributed to excited vibrational levels of the 5*s* electronic state. The 5*s* state assigned here could be related to an absorption feature that has previously been observed in different solvents between 5.51 and 5.66 eV.^[^ ^68^, ^69^ ^]^

**Table 5:** TDDFT results for selenofenchone: vertical electronic singlet excitation energies *E*_calc_, calculated energies $E_{\text{calc}}^{*}$ shifted such that the 5*s* energy matches the measured value, experimental state energies *E*_exp_, two‐photon excitation cross sections for linearly (circularly) polarized light *σ*_lin_ (*σ*_circ_) and oscillator strengths *f*. Parentheses mark a tentative assignment; italics in $E_{\text{calc}}^{*}$ mark states with significant valence character.

TDDFT selenofenchone						
Electronic transitions	*E*_calc_ [eV]	*E*_calc_* [eV]	*E*_exp_ [eV]	*σ*_lin_ [10^−50^ cm^4^ s]	*σ*_circ_ [10^−50^ cm^4^ s]	*f*
$n_{y}\to \pi_{x}^{*}$	2.298	*2.312*		0.0000	0.0000	0.0000
$\pi_{x}\to \pi_{x}^{*}$	4.866	*4.880*		0.0082	0.0003	0.2391
*n*_*y*_ → 5*s*	5.290	5.304	5.304	0.0235	0.0352	0.0394
$\sigma_{-2}\to \pi_{x}^{*}$	5.445	*5.459*		0.0008	0.0011	0.0057
*n*_*y*_ → 5*p*_*z*_	5.699	5.713		0.0062	0.0093	0.0036
*n*_*y*_ → 5*p*_*x*_	5.896	5.910	5.91	0.0172	0.0171	0.0003
*n*_*y*_ → 5*p*_*y*_	5.925	5.939		0.0404	0.0097	0.0051
$\pi_{y}\to \pi_{x}^{*}$ (84.42%), *n*_*y*_ → 4*d*_(*xx*)_ (15.05%)	6.149	6.163		0.0021	0.0029	0.0005
*n*_*y*_ → 4*d*_*xx*−*zz*_	6.162	6.176	6.16	0.0077	0.0114	0.0030
*n*_*y*_ → 4*d*_(*yy*)_	6.348	6.362		0.0055	0.0071	0.0035
*n*_*y*_ → 4*d*_*yz*+*xy*_	6.382	6.396	6.39	0.0773	0.0145	0.0052
*n*_*y*_ → 4*d*_*xy*−*yz*_	6.391	6.405		0.0511	0.0146	0.0061
*n*_*y*_ → 4*d*_*xz*_	6.442	6.456		0.0087	0.0130	0.0004
*n*_*y*_ → 6*s*	6.487	6.501		0.0011	0.0008	0.0060
$\sigma_{-3}\to \pi_{x}^{*}$	6.536	6.55		0.0159	0.0158	0.0130
*n*_*y*_ → 6*p*_*z*_	6.555	6.569		0.0021	0.0030	0.0019
*n*_*y*_ → 6*p*_*x*_	6.623	6.637		0.0017	0.0020	0.0000
$\sigma_{-3}\to \pi_{x}^{*}$	6.635	6.649		0.0087	0.0035	0.0000
*n*_*y*_ → 6*p*_*y*_	6.642	6.656		0.0062	0.0025	0.0014
$\pi_{x}\to 6s$ (77.7%), *n*_*y*_ → 4*f*_*zzz*_ + 7*p*_(*z*)_ (19.41%)	6.695	6.709		0.0086	0.0122	0.0468
*n*_*y*_ → 4*f*_*zzz*_ + 7*p*_*z*_ (79.64%), $\pi_{x}\to 7s$ (19.82%)	6.699	6.713		0.0037	0.0055	0.0148
$\sigma_{-4}\to \pi_{x}^{*}$	6.747	6.761		0.0124	0.0132	0.0202
*n*_*y*_ → 5*d*_(*xx*)_ + 4*f*_*xxz*−*yyz*_	6.799	6.813		0.0052	0.0077	0.0011
*n*_*y*_ → 4*f*_(*xxx*)_ + 5*d*_(*xx*)_	6.834	6.848		0.0016	0.0023	0.0081
*n*_*y*_ → 7*p*_(*x*)_ + 4*f*_(*yyy*−3*xxy*)_	6.848	6.862		0.0032	0.0038	0.0019
*n*_*y*_ → 6*d*_*yz*_	6.862	6.876		0.0122	0.0035	0.0010
*n*_*y*_ → 6*d*_*xy*_	6.875	6.889		0.0064	0.0061	0.0048
*n*_*y*_ → 6*d*_*yy*−*zz*_	6.886	6.900		0.0002	0.0002	0.0025
*n*_*y*_ → 6*d*_(*yz*)_	6.895	6.909		0.0035	0.0012	0.0002
*n*_*y*_ → 6*d*_*xz*_	6.926	6.940		0.0037	0.0054	0.0032

**Table 6:** CC2 results for selenofenchone: vertical electronic singlet excitation energies *E*_calc_, calculated energies $E_{\text{calc}}^{*}$ shifted such that the 5*s* energy matches the measured value, experimental state energies *E*_exp_, and oscillator strengths *f*. Parentheses mark a tentative assignment; italics in $E_{\text{calc}}^{*}$ mark states with significant valence character.

CC2 selenofenchone				
Electronic transitions	*E*_calc_ [eV]	*E*_calc_* [eV]	*E*_exp_ [eV]	*f*
$n_{y}\to \pi_{x}^{*}$	2.228	*2.276*		0.0000
$\pi_{x}\to \pi_{x}^{*}$	5.042	*5.090*		0.2578
*n*_*y*_ → 5*s*	5.256	5.304	5.304	0.0268
*n*_*y*_ → 5*p*_*z*_	5.569	5.617		0.0167
$\sigma_{1}\to \pi_{x}^{*}$	5.621	*5.669*		0.0139
*n*_*y*_ → 5*p*_*x*_	5.756	5.804	5.91	0.0002
*n*_*y*_ → 5*p*_*y*_	5.777	5.825		0.0029
*n*_*y*_ → 4*d*_*xx*−*zz*_	6.006	6.054	6.16	0.0006
*n*_*y*_ → 4*d*_*yz*_	6.195	6.243		0.0035
*n*_*y*_ → 4*d*_*yy*_	6.203	6.251		0.0013
*n*_*y*_ → 4*d*_*yz*+*xy*_	6.231	6.279	6.39	0.0017
*n*_*y*_ → 4*d*_*xy*−*yz*_	6.264	6.312		0.0012
*n*_*y*_ → 6*s*	6.356	6.404		0.0093
$\pi_{y}\to \pi_{x}^{*}$	6.373	6.421		0.0017
*n*_*y*_ → 6*p*_*z*_	6.497	6.545		0.0006
*n*_*y*_ → 6*p*_*x*_	6.575	6.623		0.0001
*n*_*y*_ → 6*p*_*y*_	6.595	6.643		0.0029
*n*_*y*_ → 4*f*_*zzz*_ + *p*_(*z*)_	6.616	6.664		0.0016
$\pi_{3}\to \pi_{x}^{*}$	6.633	*6.681*		0.0234
$\sigma_{2}\to \pi_{x}^{*}$	6.708	*6.756*		0.0004

**Figure 10:**
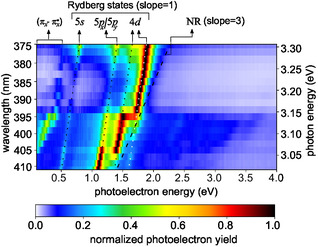
Multiphoton photoelectron spectra of selenofenchone. Each row in the figure represents a measurement at a different central wavelength. All measurements used a pulse duration of 0.4 ps and linear polarization. In each row, the signal is normalized to its maximum. Lines indicate linear scaling that can be attributed to different processes: 2 + 1 REMPI through the labeled Rydberg states yields a slope of one, while NR multiphoton ionization is associated with a slope of three. The corresponding second‐order Legendre coefficients are presented in Figure A4 in the Appendix.

To determine the adiabatic *I*_*P*_ and to identify more states, we measured multiphoton photoelectron spectra for 19 different central wavelengths ranging from 375 to 411 nm, which is shown in **Figure** **10**. In analogy to the case of fenchone (Section 4.1), we identify lines with a slope of one as associated with Rydberg states via 2 + 1 REMPI. The line originating at 0.5 eV corresponds to the 5*s* Rydberg state of selenofenchone. Fit by Equation (3), using the 5*s* Rydberg energy from ns REMPI (*E*_Ryd_ = 5.304 eV) results in an adiabatic ionization energy $I_{P}^{\text{selen}}=(7.81\pm 0.03)\;\text{eV}$ which is in close agreement with the vertical *I*_*P*_ of 7.898 eV determined by the TDDFT calculation.

**Figure A4:**
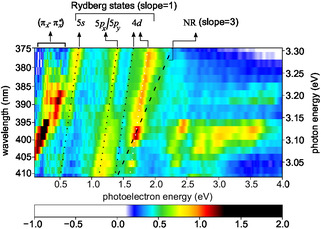
Ratio of second‐order Legendre coefficient *c*_2_ to the total signal *c*_0_ for the wavelength‐dependent multiphoton photoelectron spectra of selenofenchone presented in Figure 10.

With the knowledge of *I*_*P*_, we can use fits by Equation (3) to the other lines with a slope of one in Figure 10 and **A4** to determine the energies *E*_Ryd_ of further Rydberg states of selenofenchone. We attribute the line originating around 1 eV to a 5*p* state and obtain its energy (5.91 ± 0.05) eV. Around this energy, the TDDFT calculations (see Table 5) yield 5*p*_*x*_ and 5*p*_*y*_ states with an energy separation of just 0.05 eV, below our experimental resolution. We could not observe the lower‐lying 5*p*_*z*_ state in our multiphoton photoelectron spectra, which is in line with the significantly lower linear TPA cross section in the TDDFT calculations. The CC2 model also predicts a small energy separation between the 5*p*_*x*_ and the 5*p*_*y*_ states, but all 5*p* Rydberg states are ≈0.1 eV lower than the TDDFT values and the measured energy (see Table 6).

Figure 10 contains two lines originating around 1.3 and 1.6 eV, which show a threshold behavior near 388 and 379 nm, respectively. Fit of the lower‐lying state by Equation (3) results in an energy of (6.16 ± 0.06) eV, which matches the 4*d*_*xx*−*zz*_ state in the TDDFT calculation (Table 5). The energy determined by the CC2 model is about 0.15 eV lower than in the experiment, matching the general trend observed above (Table 6). For the higher‐lying state, we obtained the energy of (6.39 ± 0.06) eV. At this energy, the TDDFT model calculates the 4*d*_*yz*+*xy*_ and 4*d*_*xy*−*yz*_ states, which again have a slightly lower energy in the CC2 calculation. The assignments of the states at (6.16 ± 0.06) and (6.39 ± 0.06) eV as 4*d* states are supported by our quantum defect calculations, but the assignment of *m*‐levels has to be considered as tentative. Beyond 2 eV, the photoelectron spectra show only weak and featureless contributions, which we are unable to identify.

**Figure 11:**
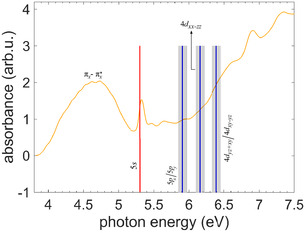
Single‐photon VUV absorption spectrum of gas‐phase selenofenchone measured with a deuterium lamp. The vertical red line shows the 5*s* Rydberg state from ns 2 + 1 REMPI spectrum where the energy uncertainty is smaller than the width of the line. Vertical blue lines indicate other Rydberg states from multiphoton photoelectron spectra, with their uncertainties shaded in grey.

Aside from the Rydberg states following the Δ*v* = 0 propensity rule, Figure 10 features a photoelectron signal below the 5*s* Rydberg state (<0.5 eV). This feature is very prominent in the map of the second‐order Legendre coefficient **Figure** **A4**. It does not show a linear dependence on photon energy, which implies that its origin is neither NR multiphoton ionization nor REMPI through a Rydberg state. Instead, we suspect a REMPI process via an intermediate ($\pi_{x}\to \pi_{x}^{*}$) valence‐excited state, which is predicted by both our calculations and observed in our single‐photon VUV absorption spectrum described below (**Figure** **11**). Interestingly, while the ($\pi_{x}\to \pi_{x}^{*}$) state is seen in the UV absorption spectrum of thiofenchone (see Figure 8) and should also exist for fenchone, ionization via this state is not observed in the multiphoton photoelectron spectra of these two molecules.

In Figure 11, the determined Rydberg state energies are compared with the measured single‐photon VUV gas‐phase absorption spectrum of selenofenchone. The broad feature starting around 3.9 eV and peaking around 4.6 eV can be assigned to the valence‐excited $\pi_{x}\to \pi_{x}^{*}$ state that previously has been reported to display an absorption maximum at similar photon energies in various solvents.^[^ ^68^, ^69^ ^]^ As in the case of thiofenchone, the vertical transition energies obtained from the calculations (TDDFT: 4.866 eV and CC2: 5.042 eV, see Table 5 and 6) overestimate the peak value around 4.6 eV. The narrow peak at about 5.3 eV matches the 5*s* Rydberg state. Above this energy, there are no clear peaks in the spectrum. However, the absorption gradually increases from about 5.8 eV. The previously assigned 5*p* and 4*d* states are in this region of increasing absorption, for which our calculations predict the contributions of multiple states.

### 4.4 Scaling of Excited State Energies in Chalcogenofenchones

**Figure 12:**
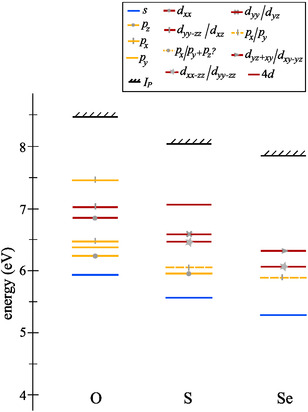
Experimentally determined Rydberg state energies and ionization energies *I*_*P*_ of fenchone (O), thiofenchone (S), and selenofenchone (Se). The line colors refer to the angular momentum of the Rydberg state (blue: *l* = 0, yellow: *l* = 1, red: *l* = 2).

We inspect general trends in the measured and calculated excited state energies of chalcogenofenchones. **Table** **7** and **Figure** **12** summarize the measured Rydberg state energies and ionization energies in fenchone, thiofenchone, and selenofenchone. For all three molecules, we could assign at least one *n*_min_ *l* Rydberg state for each angular momentum quantum number *l* = {0, 1, 2}. Here, *n*_min_ is the minimum accessible principal quantum number for each molecule and angular momentum. We see a general trend of bathochromic (red) shifts: The state energies decrease with increasing atomic number of the chalcogen.

**Table 7:** Experimentally determined electronic state energies *E*_exp_ and ionization energies *I*_P_ from spectroscopic experiments and the TDDFT assignments of electronic states for fenchone, thiofenchone, and selenofenchone. Parentheses mark a tentative assignment.

Fenchone		Thiofenchone		Selenofenchone	
Electronic states	*E*_exp_ [eV]	Electronic states	*E*_exp_ [eV]	Electronic states	*E*_exp_ [eV]
3*s*	5.954	4*s*	5.573	5*s*	5.304
3*p*_*z*_	6.306	4*p*_*z*_	5.99		
3*p*_*y*_	6.396	4_*px*_/4_*py*_	6.11	5_*px*_/5_*py*_	5.91
3*p*_*x*_	6.464				
3*d*_*xx*_	6.85	3*d*_*xx*−*zz*_	6.46	4*d*_*xx*−*zz*_	6.16
3*d*_*yy*−*zz*_/3*d*_*xz*_	7.04	3*d*_*yy*_/3*d*_*yz*_	6.62	4*d*_*yz*_ + xy/4*d*_*xy*−*yz*_	6.39
4*p*_*x*_	7.46	4*d*	7.14		
*I*_*P*_	8.48	*I*_*P*_	8.07	*I*_*P*_	7.81

The bathochromic shifts are slightly different depending on the angular momentum of the Rydberg states. For the *n*_min_ *s* states, the shift from fenchone to selenofenchone is 0.65 eV, while it is only 0.49 eV for the *n*_min_ *p*_*y*_ states. For the *n*_min_ *d* states, direct comparison is difficult because equivalent orbitals were only found for thiofenchone and selenofenchone. But here for the *n*_min_ *d*_*xx*−*zz*_, we get a difference of 0.3 eV, which is larger than the shifts between thiofenchone and selenofenchone for the *s* and *p* states. In addition, the difference between the ionization energies of fenchone and selenofenchone is 0.67 eV, comparable to the shift of the *s* Rydberg states.

**Figure 13:**
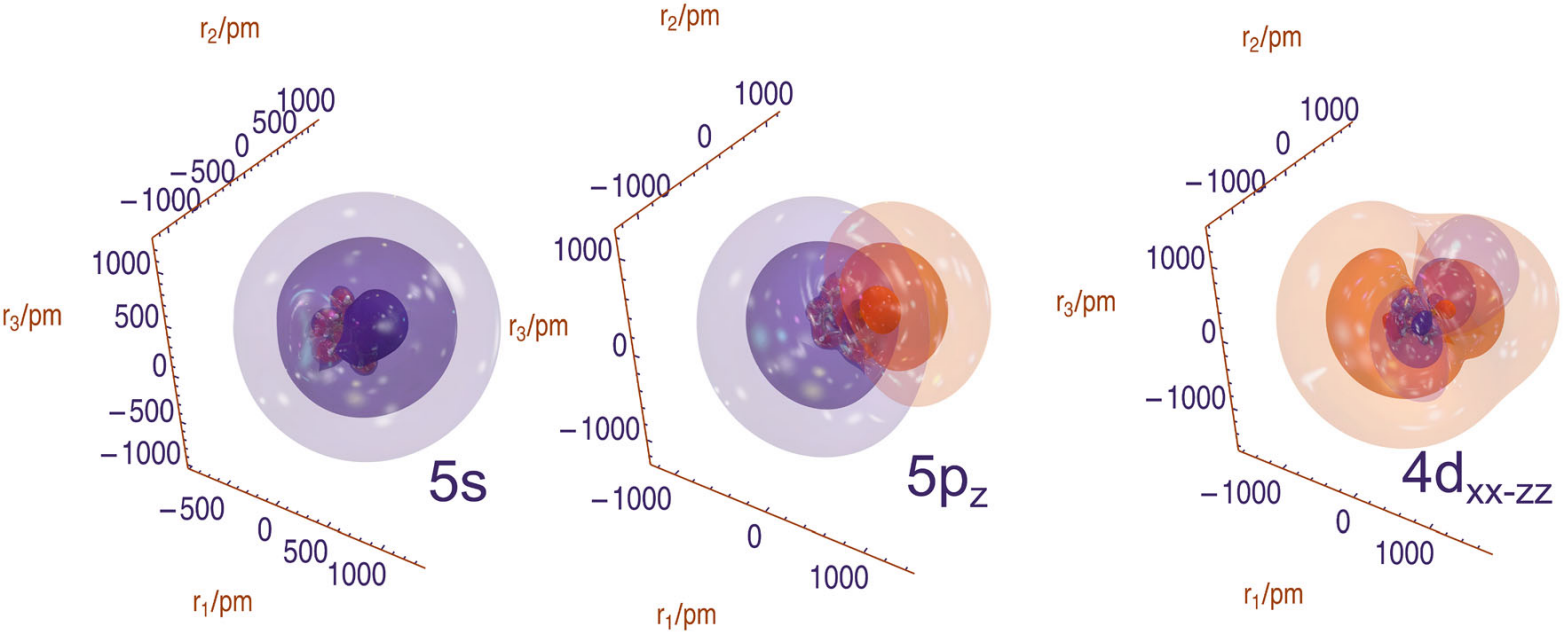
Example electron NTOs for selenofenchone with 5*s*‐, 5*p*_*z*_‐, and 4*d*_*xx*−*zz*_‐character, i.e., for the lowest energy excitations of each given *l*, showing the influence of the core region on the Rydberg‐like NTO. For each NTO, 3 nested isosurfaces are shown, with contour values scaling as ±2/(10 × 9^*n*−1^) for the *n*‐th surface and an opacity scaling of 6/(10 × 3^*n*−1^).

The trend of *l*‐dependent bathochromic shifts is well‐reproduced by both calculations when considering the shifted energy values $E_{\text{calc}}^{*}$. Angular‐momentum‐dependent shifts are expected due to the contributions of the molecular core to the Rydberg‐like state. The electron NTOs depicted in **Figure** **13** for the lowest energy *s*, *p*, *d*‐like states in selenofenchone illustrate this influence, showing regions of different phase of the orbital close to the nuclei and following the shape of the molecule instead of being purely hydrogen‐like. Additionally, the figure shows that the 4*d*_*xx*−*zz*_ orbital of selenofenchone has a significant admixture of an *s*‐function, which would further increase the influence of the structure of the molecular system due to the increased probability of the electron being in the core region.

**Figure 14:**
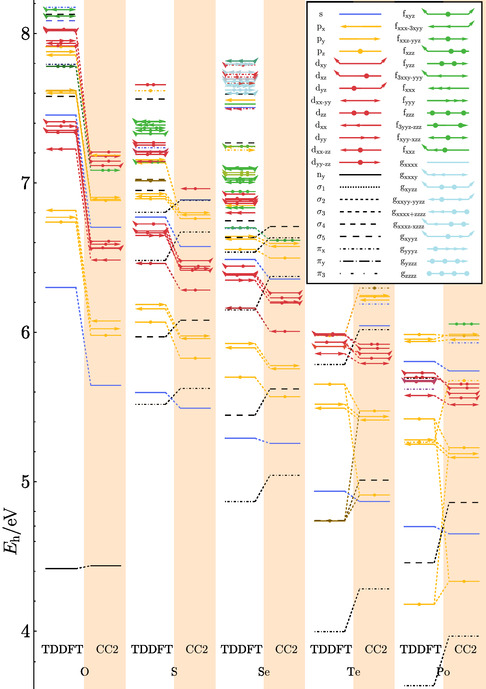
Energetic levels of excitations in the chalcogenofenchones from O to Po with symbolic indication of *l*, *m* quantum‐number related characteristics of the Rydberg‐like NTOs. TDDFT‐level results are compared to those at CC2 level, with lines connecting states with equivalent characteristics. The colors and symbols are explained in the legend on the top right.

For the experimentally identified Rydberg states, the shifting energies as a function of chemical substitution are reproduced by our theoretical models. However, they can give a broader overview, as demonstrated in **Figure** **14**, which includes tellurofenchone and polonofenchone that were unavailable for the experiment. For these two molecules, the bathochromic shifts continue. In addition, Figure 14 displays that valence‐excited states shift much stronger than Rydberg states.

**Figure A5:**
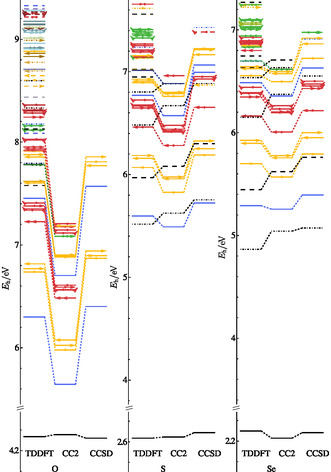
Relative energetic levels of excitations in the chalcogenofenchones from O to Se with symbolic indication of *l*, *m* quantum‐number related characteristics of the Rydberg‐like NTOs. TDDFT‐and CC2‐level results are compared to those at CCSD level, with lines connecting states with equivalent characteristics. The colors and symbols are explained in the legend in the top right of Figure 14. The cut in the energy axes removes 1.2 eV, which is the same in all three plots.

Figure 14 also helps to compare the two models: For fenchone, the Rydberg‐like excitations (nonblack in the figure) are strongly reduced in energy in CC2 compared to TDDFT. This is a known failure of the CC2 approximation, which has been shown to give less accurate results for Rydberg‐like excitations in small molecules,^[^ ^94^ ^]^ including calculations for low‐*l* excitations in fenchone.^[^ ^62^, ^63^ ^]^ While the $n_{y}\to \pi_{x}^{*}$ valence excitation energies (not shown in the Figure but cf. **Figure** **A5** and Table 1, 2, 3, 4, 5, 6 and **A1**, **A2**, **A3**, **A4** **–A** **4**) are similar in both models for all chalcogenofenchones, a different trend can be observed for the valence excitations from energetically lower‐lying occupied states: While the energy differences between the methods decrease with increasing chalcogen atomic number for the Rydberg‐like states, this difference increases for the valence‐excitations not stemming from *n*_*y*_ (see Figure A5 in the Appendix for a comparison to calculations on the CCSD level for O, S, and Se).

The failure of CC2 for the Rydberg‐like states can be attributed to the approximations involved.^[^ ^94^ ^]^ TDDFT, on the other hand, should generally only be used for low‐lying valence states,^[^ ^95^ ^]^ so a larger difference between the methods for higher‐energy valence states, which we observe in Figure A5, is not unexpected. However, the fact that the energy differences between the methods for the Rydberg states decrease and increase for the valence states for the chalcogenofenchone series shows that further benchmarking of the quality of the methods needs to include heavier systems.

When comparing the performance of the two models to the experiment, we observe that the TDDFT and CC2 reproduce the energy differences between Rydberg states with a similar quality depending on the specific selection of relative states, especially for thiofenchone and selenofenchone.

## 5 Femtosecond REMPI‐PECD of Chalcogenofenchones

**Figure 15:**
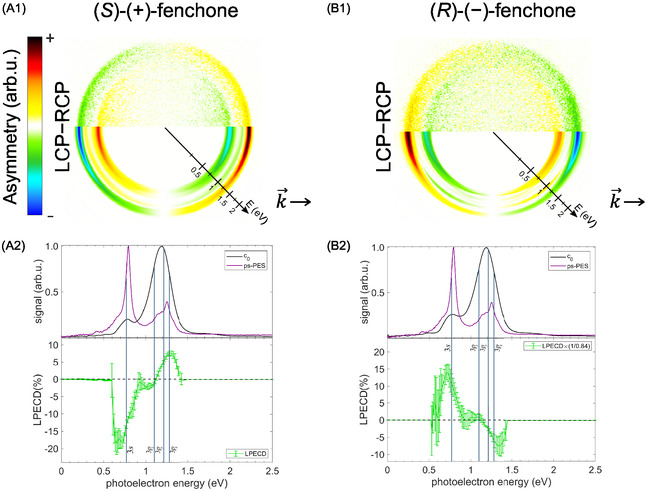
Femtosecond 2 + 1 REMPI‐PECD of fenchone enantiomers at a central wavelength of 376 nm. In the upper row, A1,B1) show the antisymmetrized PECD images where the laser pulses propagate from left to right. The upper half of each PECD image is raw, and the lower half is Abel inverted. In the lower row, A2,B2) show the total photoelectron signal *c*_0_ in the upper panels in black (for comparison, the corresponding ps photoelectron spectrum from Figure 4 in purple) and the measured LPECD values as a function of photoelectron energy in the lower panels. For the LPECD, statistical errors are shown; the uncertainties in the photoelectron spectra are much smaller. Vertical lines indicate the photoelectron energies corresponding to the Rydberg states that were identified in Section 4.1. Corresponding odd‐order Legendre coefficients are presented in Figure A6 in the Appendix.

**Figure 16:**
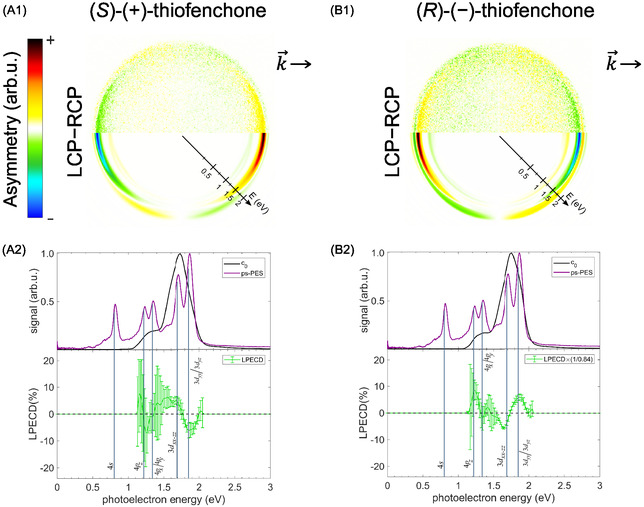
Femtosecond 2 + 1 REMPI‐PECD of thiofenchone enantiomers at a central wavelength of 376 nm. In the upper row, A1,B1) show the antisymmetrized PECD images where the laser pulses propagate from left to right. The upper half of each PECD image is raw, and the lower half is Abel inverted. In the lower row, A2,B2) show the photoelectron signal *c*_0_ in the upper panels in black (for comparison, the corresponding ps photoelectron spectrum from Figure 7 in purple) and the measured LPECD values as a function of photoelectron energy in the lower panels. For the LPECD, statistical errors are shown; the uncertainties in the photoelectron spectra are much smaller. Vertical lines indicate the photoelectron energies corresponding to the Rydberg states that were identified in Section 4.2. Corresponding odd‐order Legendre coefficients are presented in Figure A7 in the Appendix.

**Figure 17:**
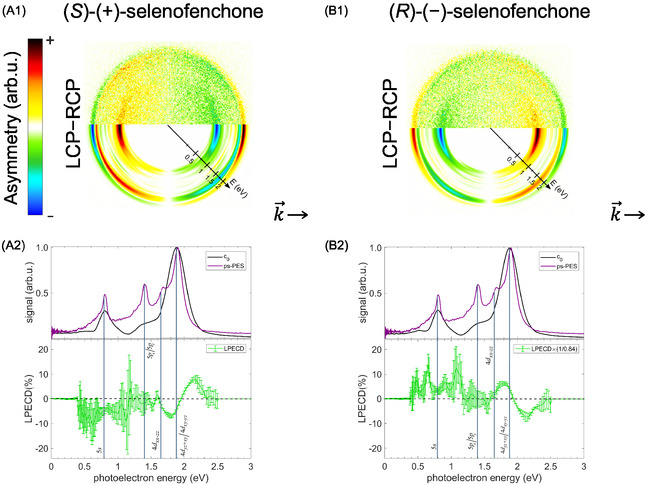
Femtosecond 2 + 1 REMPI‐PECD results of selenofenchone at a central wavelength of 376 nm. In the upper row, A1,B1) show the antisymmetrized PECD images where the laser pulses propagate from left to right. The upper half of each PECD image is raw, and the lower half is Abel inverted. In the lower row, A2,B2) show the photoelectron signal *c*_0_ in the upper panels in black (for comparison, the corresponding ps photoelectron spectrum from Figure 10 in purple) and the measured LPECD values as a function of photoelectron energy in the lower panels. For the LPECD, statistical errors are shown; the uncertainties in the photoelectron spectra are much smaller. Vertical lines indicate the photoelectron energies corresponding to the Rydberg states that were identified in Section 4.3. Corresponding odd‐order Legendre coefficients are presented in Figure A8 in the Appendix.

In **Figure** **15**, **16**, **17**, we present experimental femtosecond 2 + 1 REMPI‐PECD results for both enantiomers of fenchone, thiofenchone, and selenofenchone. All data was obtained at a central wavelength of 376 nm, i.e., very close to the upper end of the multiphoton photoelectron and second‐order Legendre coefficient maps presented above. The fully compressed, 25 fs duration laser pulses minimize the potential influence of molecular dynamics, such as internal conversion and vibration, during the REMPI‐PECD process.^[^ ^36^, ^65^ ^]^ The intensity was estimated to 3×10^12^ Wcm^−2^ for all measurements. In each figure, the results in column (A) were obtained with the (*S*)‐(+) enantiomer, while the results in column (B) were obtained with the (*R*)‐(−) enantiomer. In the upper row of each figure, panels (A1) and (B1) show the antisymmetrized PECD images with a horizontal laser propagation direction from left to right. The upper half of each PECD image results from the subtraction of raw photoelectron images for left and right circularly polarized light, while the lower half results from the Abel‐inverted photoelectron momentum distributions. In the lower row of each figure, panels (A2) and (B2) show the total photoelectron signal represented by the Legendre coefficient *c*_0_ as a function of photoelectron energy in black (upper half). In both panels, summed *c*_0_ spectra of left and right circularly polarized light are presented. In the lower half of the lower row, measured LPECD values are plotted as a function of photoelectron energy. These graphs are obtained from the odd‐order Legendre coefficients of the Abel inversion according to LPECD. Vertical lines indicate the photoelectron energies associated with the respective resonant Rydberg states identified in Section 4. The direct connection between the PECD images in the upper rows and the LPECD spectra in the lower rows should be noted: Peaks in the latter appear as rings in the former.

First, we compare the photoelectron spectra, measured with 25 fs duration laser pulses with circular polarization, with the respective multiphoton photoelectron spectra obtained for linearly polarized pulses with 0.4 ps duration, at a central wavelength of 375 nm, as contained in Figure 4, 7, and 10. Therefore, these spectra are reproduced as purple lines in panels (A2) and (B2) of Figure 15, 16, A4. For fenchone (Figure 15), the signal associated with the 3*p*_*y*_ and 3*p*_*x*_ states is about five times more intense than the 3*s* signal in the femtosecond PES, while the 3*p*_*y*_/3*p*_*x*_ signal is about half of the 3*s* state signal in the picosecond PES. This behavior in fenchone has been observed in a recent study of the dependence of PECD on the pulse duration.^[^ ^65^ ^]^ The reason for this observation is a decay of the 3*p* states to the 3*s* state with a lifetime of around 100 fs. Hence, the relative yield of resonant ionization via the 3*s* state is strongly increased for the long pulses compared to the short pulses, which is what we observe here, too. For the same reason, our observation for 25 fs pulse duration features a two times stronger relative contribution of 3*p*_*y*_/3*p*_*x*_ as compared to Kastner et al.^[^ ^36^ ^]^ where the pulse duration was around 55 fs for the same wavelength.

For thiofenchone (Figure 16), the ratio of the signal associated with 4*p* over 3*d* is about 1:2 in the picosecond photoelectron spectrum, while it is about 1:5 in the femtosecond photoelectron spectra. We conclude that the lifetime of the 3*d* states is significantly shorter than the lifetime of the 4*p* states for thiofenchone. The 4*s* state, due to its presumably long lifetime, is visible in the picosecond photoelectron spectra but not in the femtosecond data of Figure 16. For selenofenchone (Figure 17), we observe a relative intensity of the 5*s*/5*p*/4*d* signals of 2:3:5 for the picosecond data and 2:1:6 for the femtosecond data. The almost constant ratio between 5*s* and 4*d* implies that either both of their lifetimes are significantly longer than the 0.4 ps pulse duration or that they are both similar. The fact that the relative 5*p* yield is significantly higher for picosecond as compared to femtosecond pulses suggests, however, that 5*s* and 4*d* decay faster than 5*p*, on a timescale shorter than the 0.4 ps pulse duration.

Next, we note that the angle‐integrated LPECD values in panels (A2) and (B2) of Figure 15, 16, A4 depend on the photoelectron energy and feature both positive and negative signs for all enantiomers and molecules. In some cases, there is a pronounced LPECD effect in the wings of the peaks in the photoelectron spectrum, where the photoelectron yield is already very small, for example, the large LPECD in fenchone associated with the 3*s* state that peaks at an ≈0.1 eV lower energy than the corresponding photoelectron signal (see Figure 15A2,B2).

**Figure A6:**
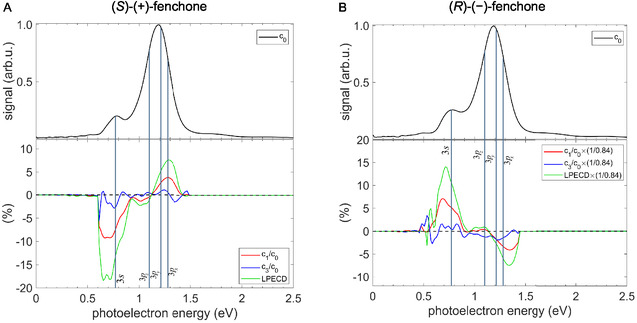
Odd‐order Legendre coefficients *c*_1_ (red) and *c*_3_ (blue), and LPECD (green) of femtosecond 2 + 1 REMPI‐PECD measurement of (A) (*S*)‐(+)‐fenchone and (B) (*R*)‐(‐)‐fenchone at a central wavelength of 376 nm in the lower panels. The upper row presents the total photoelectron spectrum represented by the Legendre coefficient *c*_0_. Vertical lines indicate the photoelectron energies corresponding to the Rydberg states that were identified in Section 4.1.

Apart from the different ratio between the 3*p* and 3*s* signals, both our photoelectron and PECD results for fenchone are in good agreement with the earlier work of Kastner et al.^[^ ^36^ ^]^ The strongest LPECD value at ≈±15% is observed for the 3*s* state, while there is a weak LPECD signal of the same sign in the 3*p*_*z*_ region. At photoelectron energies closest to the 3*p*_*x*_ state, the LPECD peaks at ≈∓7% with the opposite sign. We cannot exclude that the 3*p*_*y*_ state contributes to this LPECD peak as well. We can, however, see in panels (A1) and (B1) of Figure 15 that the two PECD contributions in the 3*p* region feature not only different signs in their LPECD but also different angular distributions: While the PECD for the 3*p*_*x*_ and 3*p*_*y*_ states has most of its signal at small angles with the light's *k*‐vector, the relative contribution at larger angles is higher for the 3*p*_*z*_ state. However, this effect is not reflected in the spectra of the odd‐order Legendre coefficients *c*_1_ and *c*_3_ that are presented in **Figure** **A6**. In most cases, the LPECD of fenchone is dominated by the *c*_1_ signal while the *c*_3_ is weak and noisy.

**Figure A7:**
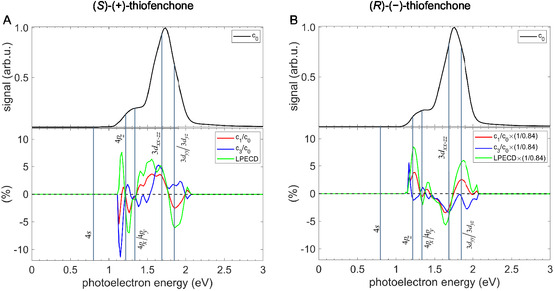
Odd‐order Legendre coefficients *c*_1_ (red) and *c*_3_ (blue), and LPECD (green) of femtosecond 2 + 1 REMPI‐PECD measurement of (A) (*S*)‐(+)‐thiofenchone and (B) (*R*)‐(‐)‐thiofenchone at a central wavelength of 376 nm in the lower panels. The upper row presents the total photoelectron spectrum represented by the Legendre coefficient *c*_0_. Vertical lines indicate the photoelectron energies corresponding to the Rydberg states that were identified in Section 4.2.

For thiofenchone, panels (A2) and (B2) of Figure 16 show that the strongest PECD contributions are at photoelectron energies associated with the 3*d* states. For the (*S*)‐(+)‐enantiomer, the 3*d*_*xx*−*zz*_ exhibits a positive LPECD peaking around 5% while the LPECD goes to ≈−4% in the photoelectron energy range associated with the 3*d*_*yy*_ or 3*d*_*yz*_ state. For the (*R*)‐(−)‐enantiomer, the signs of these PECD contributions are inverted, as can be seen in panels (B1) and (B2) of Figure 16. In addition, Figure 15A1 shows remarkably different angular distribution in the 3*d* states: The energetically lower 3*d*_*xx*−*zz*_ state mainly exhibits PECD signal close to the propagation direction of the light. In contrast, the 3*d*_*yy*_ or 3*d*_*yz*_ state mostly contributes at perpendicular angles. This difference is reflected in the spectra of the odd‐order Legendre coefficients presented in **Figure** **A7** in the Appendix: for the 3*d*_*xx*−*zz*_ state, *c*_1_ and *c*_3_ have the same sign and magnitude, while *c*_3_ almost vanishes around the 3*d*_*yy*_ and 3*d*_*yz*_ states, but has a tendency to be opposite in sign to *c*_1_. Only a very noisy LPECD signal can be observed in the energy range associated with the 4*p* states.

In selenofenchone, both the 5*s* state and the 4*d* states exhibit significant PECD. For the (*S*)‐(+)‐enantiomer (Figure 17A2), we observe an LPECD around −5% at the maximum of the 5*s* photoelectron peak and even higher LPECD values at the wings of the peak. Because of the similar redshifts in the lowest *s* Rydberg state energies and the ionization energies, 3*s* REMPI electrons in fenchone have almost the same energy as 5*s* REMPI electrons in selenofenchone. Nonetheless, the associated 5*s* LPECD in selenofenchone of 5% is lower than the 15% of the fenchone 3*s* state.

**Figure A8:**
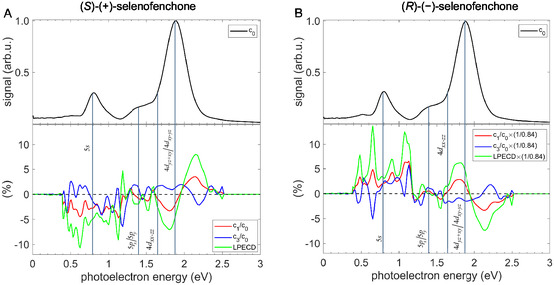
Odd‐order Legendre coefficients *c*_1_ (red) and *c*_3_ (blue), and LPECD (green) of femtosecond 2 + 1 REMPI‐PECD measurement of (A) (*S*)‐(+)‐selenofenchone and (B) (*R*)‐(‐)‐selenofenchone at a central wavelength of 376 nm in the lower panels. The upper row presents the total photoelectron spectrum represented by the Legendre coefficient *c*_0_. Vertical lines indicate the photoelectron energies corresponding to the Rydberg states that were identified in Section 4.3.

In the region of the 4*d* Rydberg states, the LPECD of selenofenchone features a sign change. For (*S*)‐(+)‐selenofenchone, the sign changes from − to + while it changes from + to − in (*S*)‐(+)‐thiofenchone. A similar difference is found in the angular distribution of the PECD: In (*S*)‐(+)‐selenofenchone, the lower‐energy part has more signal perpendicular to the light's *k*‐vector and the higher‐energy part is mainly parallel with the light propagation. These differences are not reflected in the spectra of the odd‐order Legendre coefficients displayed in **Figure** **A8** in the Appendix. In most cases, the LPECD of selenofenchone is dominated by the *c*_1_ signal while the *c*_3_ is weak and noisy. The reasons for these differences are unknown and call for further investigations. We should also note that only the negative LPECD peak of (*S*)‐(+)‐selenofenchone coincides with energies where 4*d* states have been identified by our spectroscopic experiments, namely the 4*d*_*xx*−*zz*_, 4*d*_*yz*−*xy*_, and 4*d*_*xy*−*yz*_. For the positive LPECD peak, other Rydberg states, such as 6*s* or 6*p*_*z*_, could contribute (cf. Table 5). This demonstrates that the differential detection of PECD can be more sensitive to weakly excited states than spectroscopy. For (*R*)‐(−)‐selenofenchone, the PECD behaves in the same way, but with opposite signs (see panels (A2) and (B2) of Figure 17).

## 6 Conclusion and Outlook

With our combination of spectroscopic techniques, we studied the excited states of fenchone, thiofenchone, and selenofenchone. Gas‐phase spectroscopy was performed for the first time on the latter molecule, identifying one valence‐excited and four Rydberg states as well as determining the ionization energy. Other identified states that have not been assigned previously are some 3*d* and 4*d* Rydberg states of thiofenchone and a 4*p* Rydberg state of fenchone. In addition, we improved the resolution in determining the energies of the 4*s* Rydberg state in thiofenchone and the 3*p* Rydberg states in fenchone.

The TDDFT and CC2 calculations supported the state assignment of fenchone, thiofenchone, and selenofenchone. In addition, we have calculated the electronic states of tellurofenchone and polonofenchone. With the increasing atomic number of the chalcogen, we observed different bathochromic shifts in the energies of the electronic states and the ionization energies. The valence‐excited (i.e., non‐Rydberg) states showed a stronger bathochromic shift than the Rydberg states, where the amount of shift also depends on the angular momentum quantum number (*l*).

Supplementing the spectroscopic data, we have measured 2 + 1 REMPI‐PECD using near‐ultraviolet 25 fs duration laser pulses with a central wavelength of 376 nm on fenchone, thiofenchone, and selenofenchone. For all molecules, we could identify the Rydberg states that contribute to the observed PECD signature. The strongest PECD signal in fenchone is attributed to the 3*s* Rydberg state. For thiofenchone, the PECD signal is observed only from 3*d* states, and both 5*s* and 4*d* Rydberg states dominated the REMPI‐PECD of selenofenchone.

We believe this extensive work will pave the way for further gas‐phase chiral studies and especially coherent control experiments on the series of chalcogenofenchones using a wider choice of light sources extending toward the visible spectral region.

## Conflict of Interest

The authors declare no conflict of interest.

## Supporting information

Supplementary Material

## Figures and Tables

**Figure A1:**
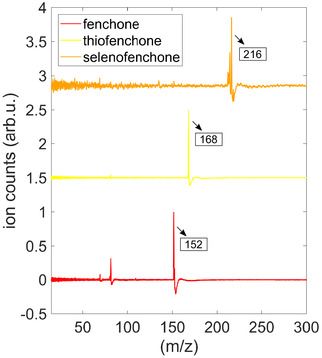
Mass spectra of fenchone, thiofenchone, and selenofenchone measured with linearly polarized 25 fs laser pulses centered around 376 nm. The pulse energy was around 3 μJ at a repetition rate of 3 kHz.

## Data Availability

The data that support the findings of this study are available from the corresponding author upon reasonable request.

## A1. Mass Spectra of Chalcogenofenchones

**Figure** **A1** displays the mass spectra of fenchone, thiofenchone, and selenofenchone using linearly polarized femtosecond laser pulses with a central wavelength of 376 nm. Pulses with the same parameters but circular polarization were used to measure PECD. We observe that the parent ion was dominant for all the molecules. Only for fenchone, a significant amount of mass signals associated with known fragments^[^ ^34^ ^]^ was found.

## A2. Second‐Order Legendre Coefficients from Multiphoton Photoelectron Spectroscopy

We show the second‐order Legendre coefficient *c*_2_ spectra obtained from multiphoton photoelectron spectroscopy for fenchone (Figure A2), thiofenchone (Figure A3), and selenofenchone (Figure A4). In all the second‐order Legendre coefficient spectra, each wavelength measurement is normalized by the respective photoelectron spectrum *c*_0_.

## A3. Theoretical Results on the CCSD Level

Figure A5 displays CCSD as a third theoretical method to compare with the TDDFT and CC2 results. It should be noted that CCSD itself is not necessarily more accurate than the other two, but the threefold comparison is useful in that it reveals trends in the methods. CC2 clearly underestimates the Rydberg state excitation energies, but the problem lessens for the heavier homologues. TDDFT, on the other hand, deviates more from CCSD than CC2 does for the valence states for excitations from orbitals energetically below the HOMO *n*_*y*_.

## A4. Theoretical Results for Tellurofenchone and Polonofenchone

Table A1 and A2 present the calculated energies and oscillator strengths for tellurofenchone, while Table A3 and A4 contain the same for polonofenchone. Table A1 and A3 are the results of the TDDFT method and Table A2 and A4 of the CC2 method.

**Table A1:** TDDFT results for tellurofenchone: vertical electronic singlet excitation energies *E*_calc_, two‐photon excitation cross sections for linearly (circularly) polarized light *σ*_lin_ (*σ*_circ_) and oscillator strengths *f*. Parentheses mark a tentative assignment.

TDDFT tellurofenchone				
Electronic transitions	*E*_calc_ [eV]	*σ*_lin_ [10^−50^ cm^4^ s]	*σ*_circ_ [10^−50^ cm^4^ s]	*f*
$n_{y}\to \pi_{x}^{*}$	1.823	0.0000	0.0000	0.0000
$\pi_{x}\to \pi_{x}^{*}$	3.996	0.0067	0.0001	0.2328
*n*_*y*_ → 6*p*_*z*_ (59.07%), $\sigma_{-2}\to \pi_{x}^{*}$ (40.49%)	4.738	0.0169	0.0251	0.0147
*n*_*y*_ → 6*s*	4.936	0.0064	0.0095	0.0362
*n*_*y*_ → 6*p*_*x*_	5.491	0.0386	0.0402	0.0004
*n*_*y*_ → 6*p*_*y*_	5.518	0.1329	0.0320	0.0085
*n*_*y*_ → 6*p*_*z*_ + 5*d*_*zz*_	5.652	0.0009	0.0013	0.0094
$\sigma_{-4}\to \pi_{x}^{*}$	5.785	0.0020	0.0015	0.0005
*n*_*y*_ → 5*d*_*xx*−*yy*_	5.857	0.0267	0.0397	0.0174
*n*_*y*_ → 5*d*_*xy*_ (84.09%), $\pi_{x}\to 7p_{z}$ (15.51%)	5.905	0.0260	0.0357	0.0123
*n*_*y*_ → 5*d*_*yz*_	5.933	0.1477	0.0242	0.0202
$\pi_{x}\to 7p_{z}(+s)$ (81.5%), *n*_*y*_ → 5*d*_*yy*+*zz*_ (18.16%)	5.977	0.0253	0.0378	0.0122
*n*_*y*_ → 5*d*_*zz*_	5.980	0.0073	0.0109	0.0027
*n*_*y*_ → 5*d*_*xz*_	5.990	0.0108	0.0154	0.0009

**Table A2:** CC2 results for tellurofenchone: vertical electronic singlet excitation energies *E*_calc_, and oscillator strengths *f*.

CC2 tellurofenchone		
Electronic transitions	*E*_calc_ [eV]	*f*
$n_{y}\to \pi_{x}^{*}$	1.680	0.0000
$\pi_{x}\to \pi_{x}^{*}$	4.282	0.2437
*n*_*y*_ → 6*s* + 6*p*_*z*_	4.867	0.0412
*n*_*y*_ → 6*p*_*z*_ + 6*s*	4.911	0.0070
$\sigma_{1}\to \pi_{x}^{*}$	5.010	0.0059
*n*_*y*_ → 6*p*_*x*_	5.413	0.0003
*n*_*y*_ → 6*p*_*y*_	5.436	0.0050
*n*_*y*_ → 6*p*_*z*_ + 5*d*_*zz*_	5.473	0.0110
*n*_*y*_ → 5*d*_*xx*−*yy*_	5.791	0.0135
*n*_*y*_ → 5*d*_*yz*_	5.826	0.0147
*n*_*y*_ → 5*d*_*xy*_	5.859	0.0083
*n*_*y*_ → 5*d*_*xz*_	5.893	0.0023
*n*_*y*_ → 5*d*_*zz*_	5.920	0.0086
$\pi_{y}\to \pi_{x}^{*}$	6.017	0.0005
*n*_*y*_ → 7*s*	6.044	0.0063
$\pi_{x}\to 6s$	6.189	0.0791
*n*_*y*_ → 7*p*_*x*_	6.216	0.0011
*n*_*y*_ → 7*p*_*z*_	6.239	0.0062
*n*_*y*_ → 7*p*_*y*_	6.246	0.0033

**Table A3:** TDDFT results for polonofenchone: vertical electronic singlet excitation energies *E*_calc_, two‐photon excitation cross sections for linearly (circularly) polarized light *σ*_lin_ (*σ*_circ_) and oscillator strengths *f*.

TDDFT polonofenchone				
Electronic transitions	*E*_calc_ [eV]	*σ*_lin_ [10^−50^ cm^4^ s]	*σ*_circ_ [10^−50^ cm^4^ s]	*f*
$n_{y}\to \pi_{x}^{*}$	1.674	0.0001	0.0001	0.0000
$\pi_{x}\to \pi_{x}^{*}$	3.640	0.0066	0.0000	0.2097
*n*_*y*_ → 7*p*_*z*_	4.179	0.0260	0.0391	0.0236
$\sigma_{-2}\to \pi_{x}^{*}$	4.457	0.0016	0.0020	0.0009
*n*_*y*_ → 7*s*	4.700	0.0076	0.0114	0.0407
*n*_*y*_ → 7*p*_*x*_	5.251	0.0579	0.0687	0.0003
$\pi_{x}\to 7p_{z}$	5.264	0.0406	0.0578	0.0147
*n*_*y*_ → 7*p*_*y*_	5.280	0.2229	0.0580	0.0089
*n*_*y*_ → 7*p*_*z*_ + 6*d*_*zz*_	5.420	0.0039	0.0057	0.0090
*n*_*y*_ → 6*d*_*xx*−*yy*_	5.577	0.0510	0.0765	0.0163
$\pi_{x}\to 8s$ (55.41%), *n*_*y*_ → 6*d*_*xy*_ (44.28%)	5.619	0.0050	0.0067	0.0155
*n*_*y*_ → 6*d*_*yz*+*xy*_ (83.06%), $\pi_{x}\to 8s$ (16.46%)	5.669	0.1445	0.0356	0.0439
*n*_*y*_ → 6*d*_*xy*−*yz*_ (74.43%), $\pi_{x}\to 8s$ (24.66%)	5.677	0.0673	0.0384	0.0598
$\sigma_{-4}\to \pi_{x}^{*}$	5.692	0.0051	0.0050	0.0015
*n*_*y*_ → 6*d*_*zz*_	5.698	0.0048	0.0071	0.0062
*n*_*y*_ → 6*d*_*xz*_	5.728	0.0145	0.0205	0.0006
*n*_*y*_ → 8*s*	5.805	0.0052	0.0074	0.0145
*n*_*y*_ → 8*p*_*x*_	5.938	0.0055	0.0080	0.0000
*n*_*y*_ → 8*p*_*y*_	5.953	0.0180	0.0110	0.0017
*n*_*y*_ → 8*p*_*z*_	5.984	0.0056	0.0084	0.0042

**Table A4:** CC2 results for polonofenchone: vertical electronic singlet excitation energies *E*_calc_, and oscillator strengths *f*.

CC2 polonofenchone		
Electronic transitions	*E*_calc_ [eV]	*f*
$n_{y}\to \pi_{x}^{*}$	1.454	0.0000
$\pi_{x}\to \pi_{x}^{*}$	3.966	0.2200
*n*_*y*_ → 7*p*_*z*_ + 7*s*	4.332	0.0398
*n*_*y*_ → 7*s* + 7*p*_*z*_	4.651	0.0260
$\sigma_{1}\to \pi_{x}^{*}$	4.860	0.0025
*n*_*y*_ → 7*p*_*x*_	5.161	0.0002
*n*_*y*_ → 7*p*_*y*_	5.187	0.0047
*n*_*y*_ → 7*p*_*z*_ + 6*d*_*zz*_	5.227	0.0115
*n*_*y*_ → 6*d*_*xx*−*yy*_	5.515	0.0185
*n*_*y*_ → 6*d*_*yz*_	5.561	0.0190
*n*_*y*_ → 6*d*_*xy*_	5.591	0.0159
*n*_*y*_ → 6*d*_*xz*_	5.627	0.0022
*n*_*y*_ → 6*d*_*zz*_	5.654	0.0046
$\pi_{x}\to 7p_{z}+7s$	5.676	0.0449
*n*_*y*_ → 8*s* + 6*d*_*zz*_	5.741	0.0146
*π*_*x*_ → 7*s* + 7*p*_*z*_	5.929	0.0389
*n*_*y*_ → 8*p*_*x*_	5.950	0.0022
*n*_*y*_ → 8*p*_*z*_	5.977	0.0071
*n*_*y*_ → 8*p*_*y*_	5.987	0.0027
*n*_*y*_ → 5f_*zzz*_	6.055	0.0166

## A5. Odd‐Order Legendre Coefficients for Femtosecond REMPI‐PECD Measurements

We present the first‐ and third‐order Legendre coefficients, *c*_1_/*c*_0_ and *c*_3_/*c*_0_, obtained from the femtosecond REMPI‐PECD measurements for all chalcogenofenchones (see Section 5). The zeroth‐order Legendre coefficient *c*_0_ corresponds to the total photoelectron spectrum.
